# Digging Up the Roots: Taxonomic and Phylogenetic Disentanglements in Corticiaceae s.s. (Corticiales, Basidiomycota) and Evolution of Nutritional Modes

**DOI:** 10.3389/fmicb.2021.704802

**Published:** 2021-08-25

**Authors:** Masoomeh Ghobad-Nejhad, Ewald Langer, Karen Nakasone, Paul Diederich, R. Henrik Nilsson, Mario Rajchenberg, James Ginns

**Affiliations:** ^1^Department of Biotechnology, Iranian Research Organization for Science and Technology, Tehran, Iran; ^2^Department of Ecology, FB 10 (Mathematics and Natural Sciences), University Kassel, Kassel, Germany; ^3^Center for Forest Mycology Research, Northern Research Station, U.S. Forest Service, Madison, WI, United States; ^4^Musée national d'histoire naturelle, Luxembourg, Luxembourg; ^5^Department of Biological and Environmental Sciences, Gothenburg Global Biodiversity Centre, University of Gothenburg, Göteborg, Sweden; ^6^Centro de Investigación y Extensión Forestal Andino Patagónico, National Research Council of Argentina (CONICET), Esquel, Argentina; ^7^Retired, Penticton, BC, Canada

**Keywords:** *Mycobernardia incrustans*, corticioid clade, *Laetisaria endoxylon*, lichenicolous basidiomycetes, lifestyle, new species, plant pathogenic fungi, systematics

## Abstract

Corticiaceae is one of the traditional families of the Agaricomycetes and served for a long time as a convenient placement for basidiomycetes with a resupinate, corticioid form of fruiting body. Molecular studies have helped to assign many corticioid fungi to diverse families and orders; however, Corticiaceae still lacks a phylogenetic characterization and modern circumscription. Here, we provide the first comprehensive phylogenetic and taxonomic revision of the family Corticiaceae based on extensive type studies and sequences of nLSU, ITS, IGS, nSSU, and mtSSU regions. Our analyses support the recognition of ten monophyletic genera in the Corticiaceae, and show that nutritional mode is not a robust basis for generic delimitations in the family. The genus *Mycobernardia* and the species *Corticium thailandicum, Erythricium vernum*, and *Marchandiomyces allantosporus* are described as new to science, and five new combinations are proposed. Moreover, ancestral character state reconstruction revealed that saprotrophy is the plesiomorphic nutritional mode in the Corticiaceae, while several transitions have occurred to diverse nutritional modes in this family. Identification keys are provided to the genera in Corticiaceae s.s. as well as to the species in *Corticium, Erythricium, Laetisaria*, and *Marchandiomyces*.

## Introduction

The family Corticiaceae has a long taxonomic history yet remains poorly known and understudied. Corticiaceae was created by Herter (1910) to encompass the vast group of aphyllophoroid fungi with a corticioid or crust form of fruiting body. These fungi were formerly referred to Thelephoraceae (Chevallier, 1826), until the name became restricted to a group of mycorrhizal fungi with variable macromorphology but quite uniform in microscopy and recognized by Patouillard (1900) as the “Série des Phylactéries.” In his important monograph, Donk (1964) defined the different families of non-gilled basidiomycetes (formerly “Aphyllophorales”) and delineated the Corticiaceae in a very wide sense to include most wood-associated basidiomycetes with a typically smooth hymenophore. This concept was widely accepted and remained largely intact until the advent of molecular taxonomy. The Thelephoraceae was used for some important fungal monographs in the USA (Burt, 1926), Australia, and New Zealand (Cunningham, 1963). One of the most eminent works on Corticiaceae (sensu Donk) includes the monograph series by John Eriksson et al.—“The Corticiaceae of North Europe” (1973–1988)—which provided detailed information and illustrations on numerous genera and species. It initiated a rapid spread in the knowledge of this group of fungi worldwide.

The advent of DNA sequencing and molecular analyses deepened our understanding of relationships among fungal groups and, thus, in their classification. Within the Agaricomycetes it was shown that the corticioid fungi were distributed in practically all the clades within the class (Larsson et al., 2004; Binder et al., 2005). Accordingly, orders were resurrected or newly described to accommodate different groups of corticioid fungi (Larsson, 2007; Binder et al., 2010; Hodkinson et al., 2014; Lücking and Moncada, 2017), and the small “corticioid clade” (Larsson et al., 2004; Binder et al., 2005, Matheny et al., 2007) was raised to be the order Corticiales K. H. Larss. (Larsson, 2007) containing a single family—Corticiaceae. All these studies used rRNA genes as molecular markers, and this was continued in the later studies in this family.

Lawrey et al. (2008) noted a remarkable nutritional diversity in the order Corticiales including saprotrophs, plant pathogens, and fungal pathogen species. A natural outcome of this observation was the question whether nutritional mode was a useful information source for generic delimitation in Corticiales. Ghobad-Nejhad et al. (2010) resurrected two more families in the Corticiales, viz. Vuilleminiaceae and Punctulariaceae, and established that the diversity of nutritional habit is concentrated in the Corticiaceae only, with the other two families being uniformly saprotrophic. A fourth Corticiales family—Dendrominiaceae—was introduced by Ariyawansa et al. (2015), consisting of saprotrophic species.

Currently, a delimitation of Corticiaceae in the modern sense is absent, and the generic boundaries in the family are vague. With diverse modes of nutrition in closely related taxa, it is also important to understand whether the nutritional lifestyle is a generic feature in this family.

The aims of this study are: (1) to analyse the circumscription and phylogenetic relationships in Corticiaceae s.s. and to resolve the delimitation of its genera, (2) to study the relationships between taxa in the genus *Corticium* (the type genus of the family), and (3) to examine the evolution of nutritional modes in the Corticiaceae.

## Materials and Methods

### Specimens and Morphology

**Figure 1:**
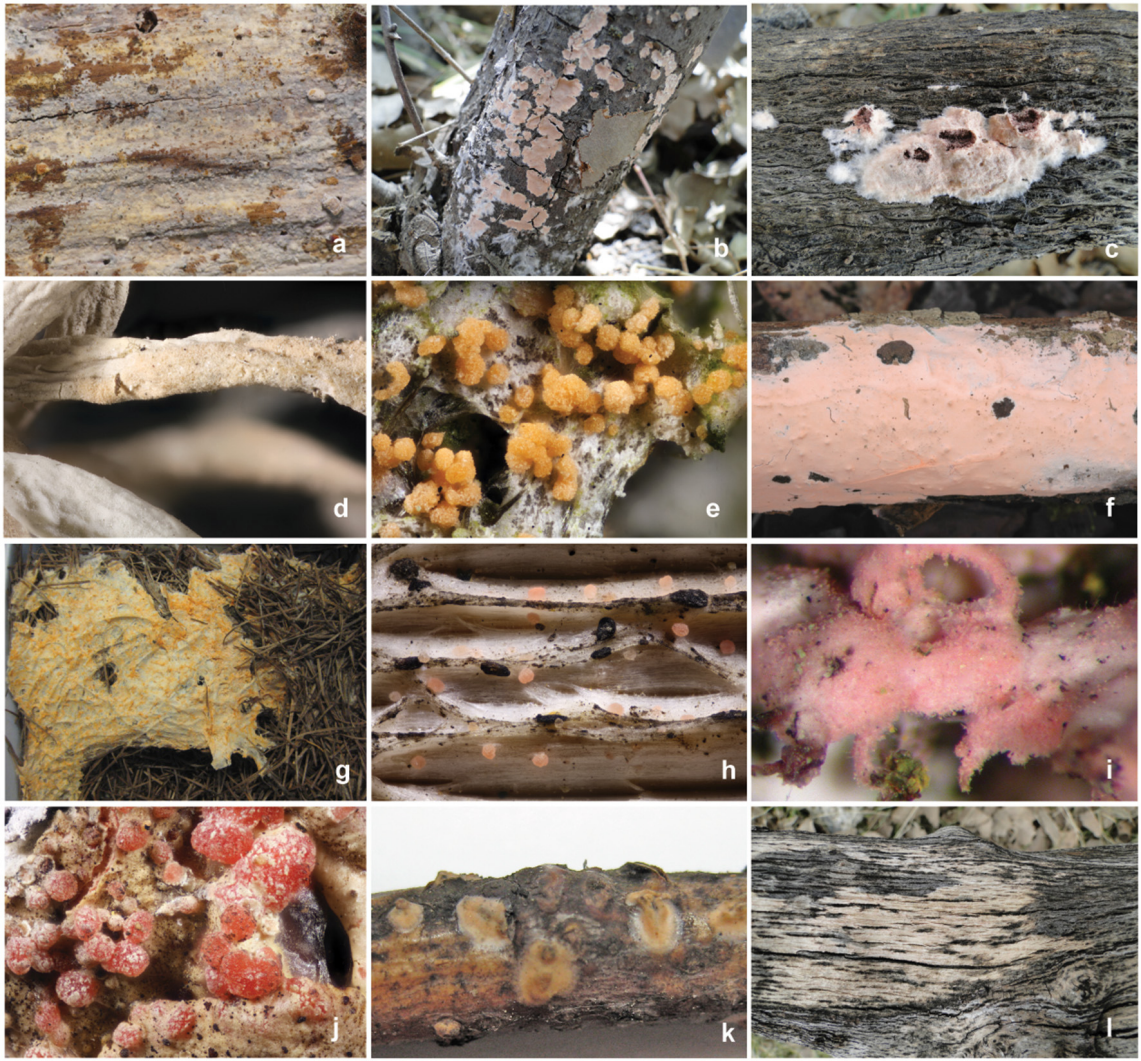
Morphological diversity in Corticiaceae s.s. **(a)** *Mycobernardia incrustans* (M. Wilhelm 61060); **(b)** *Corticium meridioroseum* (Ghobad-Nejhad 4005); **(c)** *Corticium roseum*, asexual form (syn. *C. erikssonii*) (Ghobad-Nejhad 3237); **(d)** *Corticium silviae* Holotype; **(e)** *Erythricium aurantiacum* bulbils (Diederich 17722); **(f)** *Erythricium hypnophilum* (Meyer s.n.); **(g)** *Erythricium laetum* (E. Camppo s.n.); **(h)** *Laetisaria buckii* on wood (Buck 64594); **(i)** *Laetisaria lichenicola* on *Physcia tenella* (Heklau s.n.); **(j)** *Marchandiomyces corallinus* on *Xanthoparmelia* (Heklau s.n.); **(k)** *Marchandiomyces aurantioroseus* (F119140, S); **(l)** *Marchandiomyces* sp. on wood (Ghobad-Nejhad s.n.). Photos **(a,f)** by A. Bernicchia; **(b,c,g,k,l)**: by M. Ghobad-Nejhad; **(d,e,h–j)** by P. Diederich.

Specimens were studied from various herbaria: AH, BJFC, BPI, CFMR, DAOM, H, HCFC, ICH, K, LY, MA (MA-Fungi), PC, PRM, and S. Herbaria acronyms follow Thiers (2021). Type material and other authenticated or original material was prioritized whenever available. The majority of type specimens were originally filed under *Corticium*. A complete list of examined specimens is available from the corresponding author. Representatives of Corticiaceae taxa are shown in Figure 1.

Specimens were studied under a light microscope with bright field or phase contrast optics. Squash mounts were prepared in 5% potassium hydroxide (KOH), Melzer's reagent (IKI), Cotton Blue in lactic acid (CB), and 1% phloxine. Basidiospore measurements were based on at least thirty spores per specimen. The following abbreviations are used: CB–, non-cyanophilous; IKI–, non-amyloid and non-dextrinoid; Q, length to width ratio; PP, posterior probability.

### Molecular Study

Total genomic DNA was extracted from dried fruiting bodies using the DNeasy Plant Mini Kit (QIAGEN) and, for some specimens, the rapid preparation procedure described by Izumitsu et al. (2011). The D1/D2 variable domains of the 28S rRNA (nLSU) gene, the ITS region covering ITS1, 5.8 and ITS2, the IGS1-5S domain of the nuclear ribosomal intergenic spacer region (IGS), and the mitochondrial small subunit rRNA (mtSSU) gene were amplified and sequenced using the primers listed in Table 1. Sequences were assembled and edited in MEGA6 (Tamura et al., 2013) and deposited in GenBank (Supplementary Table 1).

**Table 1:** List of primers used for PCR and sequencing in this study.

**DNA region**	**Primer pairs**	**References**
ITS	ITS1F/ITS4B	Gardes and Bruns, 1993
	ITS1/ITS4	White et al., 1990; Gardes and Bruns, 1993
	ITS1F/ITS4	White et al., 1990; Gardes and Bruns, 1993
	ITS1F/ITS2	White et al., 1990; Gardes and Bruns, 1993
	ITS3/ITS4	White et al., 1990
nLSU	LR0R/LR5	Hopple and Vilgalys, 1999
	LR0R/LR7	Hopple and Vilgalys, 1999
	NL1/NL4	O'Donnell, 1993
IGS	CNL12/5SAr	Anderson and Stasovski, 1992
mtSSU	mrSSU1/mrSSU3R	Zoller et al., 1999

### Phylogenetic Analyses

The taxa for phylogenetic analyses were sampled from the order Corticiales (Ghobad-Nejhad et al., 2010; Jayawardena et al., 2019). For a quick assessment of the taxonomic affiliation of the sequences, all newly obtained sequences were tested via NCBI blast and the taxa outside Corticiales were excluded from the analyses. An attempt was made to include sequences from type specimens, reference material, and from generic types. Four datasets were constructed: (1) a concatenated dataset of nLSU + ITS + SSU+ mtSSU to examine the outline of Corticiaceae and delimitation of its genera, (2) a combined dataset of nLSU + ITS to establish the fine-grained placement of the new species and new genus, (3) an ITS dataset covering sequences of the genus *Corticium* Pers., and (4) a combined dataset of ITS + IGS to examine the relationships of the species in the *Corticium roseum* group (Table 2).

**Table 2:** Characteristics of the datasets in this study.

					**No. of nucleotides**			
**Dataset**	**Gene partition**	**Best-fit evolutionary model**	**Outgroup**	**No. taxa**	**Total**	**Constant**	**Variable**	**Informative**
(1)	nLSU	GTR + I + G	–	–	–	–	–	–
	ITS	GTR + G	–	–	–	–	–	–
	nSSU	GTR + I + G	–	–	–	–	–	–
	mtSSU	GTR + G	–	–	–	–	–	–
	combined	–	*Gloeophyllum sepiarium*	54	3,818	2,646	486	686
(2)	nLSU	GTR + I + G	–	–	–	–	–	–
	ITS	GTR + I + G	–	–	–	–	–	–
	combined	–	*Dendrominia dryina*	78	1,459	822	213	424
(3)	ITS	GTR + I + G	*Laetisaria fuciformis*	38	686	399	79	208
(4)	ITS	GTR + I + G	–	–	–	–	–	–
	IGS	HKY + G	–	–	–	–	–	–
	combined	–	*Dendrocorticium polygonioides*	32	1,157	520	213	424

The alignments were computed in MUSCLE (Madeira et al., 2019) and optimized using Gblocks v. 0.91b (Castresana, 2000). Datasets were analyzed using MrBayes v. 3.2.7a (Ronquist et al., 2012), implementing the best-fit model of nucleotide evolution for each partition as inferred from MrModeltest 2.3 (Nylander, 2004). The datasets were analyzed using two independent runs for 20 M generations. The trees and parameters were sampled every 5,000 generations. Burn-in was set to discard 50% of samples. The majority-rule consensus tree was assembled from post-burn-in trees. The Bayesian analyses were run at the CIPRES Science Gateway (Miller et al., 2010). Maximum likelihood analyses were performed in raxmlGUI v.1.3 (Silvestro and Michalak, 2010), with the search strategy set to rapid bootstrapping and using the GTRGAMMAI model of nucleotide substitution. The number of replicates was automatically inferred through the stopping criterion (Pattengale et al., 2009). The outgroup for the four-gene dataset (dataset 1) was chosen from Russulales and Gloeophyllales, following the recent phylogenetic outline of Basidiomycota (He et al., 2019). Outgroups for the other datasets were chosen based on the results from this study.

Information on the nutritional mode of the species was assembled from the literature (endolichenic: growing asymptomatically inside lichen thalli; saprotrophic: on dead wood, leaves, debris, and other plant remnants; lichenicolous: on lichen thalli; and plant parasite: on living plants and causing disease symptoms). The ancestral state of the nutritional mode in the Corticiaceae was reconstructed using parsimony in PAUP 4.a168 (Swofford, 2021) and Bayesian inference in BayesTraits 3.0.2 (Pagel and Meade, 2017). The BayesTraits analysis used a 1 M generation Markov Chain Monte Carlo design, and the default Bayes factor test was used to assess the significance. *Waitea circinata* Warcup and P. H. B. Talbot is mostly known as plant pathogen, but has also been reported as saprotrophic on dead wood (Roberts, 2003), such that its nutritional mode was scored as uncertain. This was also done for *Waitea arvalis* and *Laetisaria agaves*.

## Results

### Molecular Study and Phylogenetic Analyses

Altogether, 85 new DNA sequences were generated in this study (Supplementary Table 1), and these were supplemented with sequences from GenBank. The oldest, successfully sequenced sample was *Erythricium vernum* (RLG-7886, CFMR) collected in 1968 (see paratypes under description of this species) (Most of the types and original material of *Galzinia* Bourdot were scanty and failed to produce satisfactory DNA in the extraction step). The characteristics of the DNA datasets including the number of taxa, number of characters, and the best-fit evolutionary model suggested by MrModeltest for each gene partition are shown in Table 2. Taxa excluded from the Corticiaceae based on our previous/current type studies are summarized in Table 3. The multiple sequence alignments were deposited in TreeBASE (accession 28108).

**Table 3:** Summary of some important taxa excluded from Corticiaceae based on our type/authentic material studies.

**Species**	**Current position**			
	**Current name**	**Family, order**	**References**	**Notes**
*Corticium cremeoalbidum* (M. J. Larsen and Nakasone) M. J. Larsen	*Punctulariopsis cremeoalbida* (M. J. Larsen and Nakasone) Ghobad-Nejhad	Punctulariaceae, Corticiales	Ariyawansa et al., 2015	–
*Corticium efibulatum* (M. J. Larsen and Nakasone) M. J. Larsen	*Punctulariopsis efibulata* (M. J. Larsen and Nakasone) Ghobad-Nejhad	Punctulariaceae, Corticiales	Ariyawansa et al., 2015	–
*Corticium hakgallae* Berk. and Broome	*Aleurocystis hakgallae* (Berk. and Broome) G. Cunn.	incertae sedis, Agaricales	Giraldo et al., 2017	Type of *Aleurocystis* Lloyd ex G. Cunn.
*Corticium subgiganteum* Berk.	*Licrostroma subgiganteum* (Berk.) P.A. Lemke	Peniophoraceae, Russulales	Giraldo et al., 2017	Type of *Licrostroma* Lemke
*Dendrominia maculata* (H. S. Jacks. and P. A. Lemke) Ghobad-Nejhad and Duhem	*Dendrominia maculata* (H. S. Jacks. and P. A. Lemke) Ghobad-Nejhad and Duhem	Dendrominiaceae, Corticiales	Ariyawansa et al., 2015	Type of *Dendrominia* Ghobad-Nejhad and Duhem
*Dentocorticium ussuricum* (Parmasto) M. J. Larsen and Gilb.	*Dentocorticium ussuricum* (Parmasto) M. J. Larsen and Gilb.	Polyporaceae, Polyporales	Li et al., 2016	Type of *Dentocorticium*
*Erythricium chaparralum* Burds. and Gilb.	*Phanerochaete chaparrala* (Burds. and Gilb.) Nakasone and Ghobad-Nejhad	–	This study	Molecular data not available
*Galzinia longibasidia* Hallenb.	*Galzinia longibasidia* Hallenb.	incertae sedis, Polyporales	Li et al., 2016	–
*Leptocorticium tenellum* Nakasone	*Leptocorticium tenellum* Nakasone	incertae sedis, Russulales	Li et al., 2016	–

**Figure 2:**
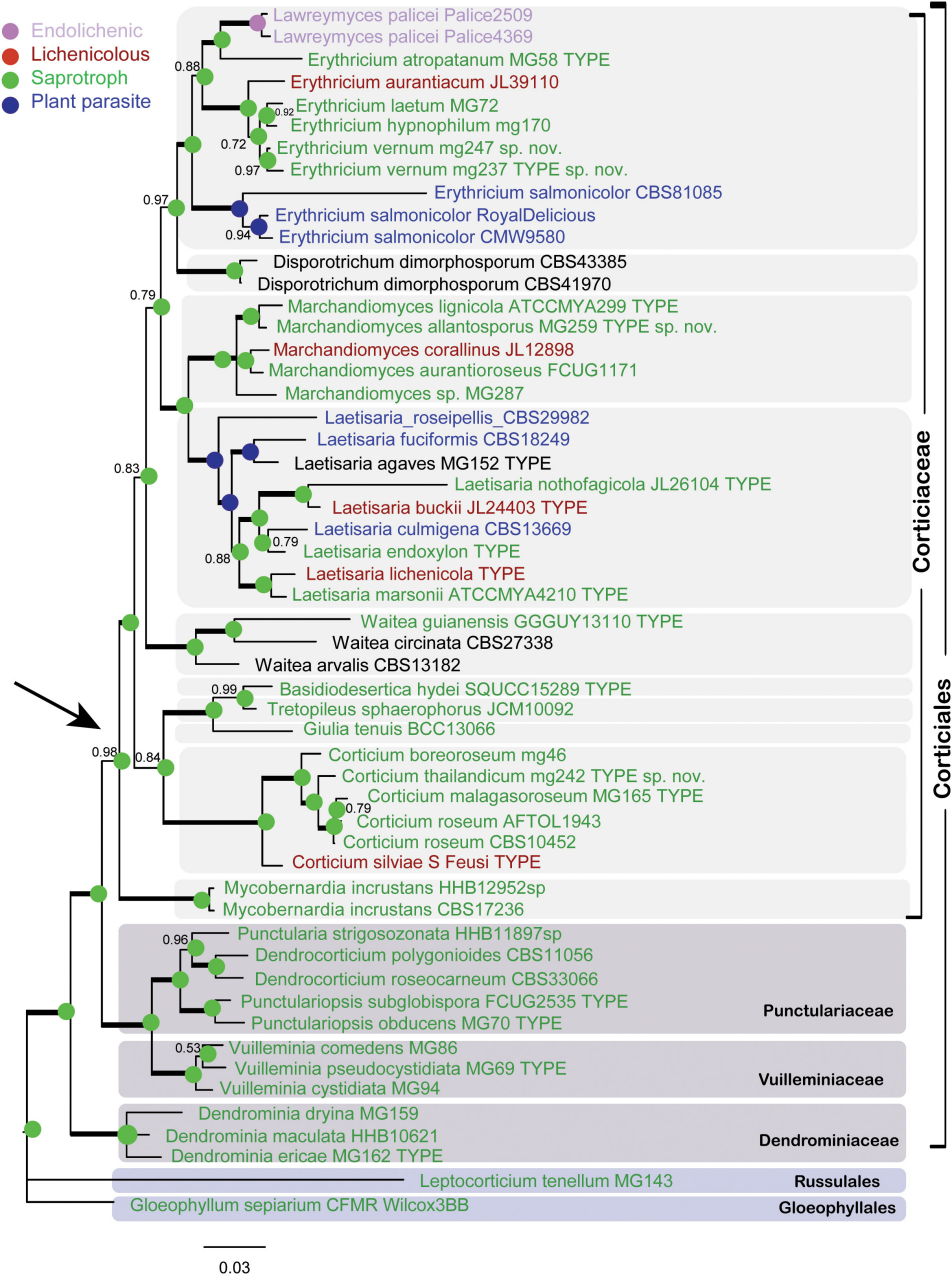
Bayesian phylogram of the concatenated nLSU-ITS-SSU-mtSSU dataset for Corticiaceae. Clades for the Corticiaceae genera are marked with light gray boxes. Branches in bold have the posterior probability = 1.00. Taxa are colored according to their nutritional mode (taxa names in black have uncertain nutritional habit). The colored nodes show the ancestral character state inferred from parsimony analyses. The tree is rooted with *Gloeophyllum sepiarium*. The scale bar represents the expected changes per site.

The Bayesian phylogram for the combined dataset of nLSU + ITS + SSU + mtSSU (dataset 1) is shown in Figure 2. The phylogram depicts an overall topology of the order Corticiales, with the four families Corticiaceae, Punctulariaceae, Vuilleminiaceae, and Dendrominiaceae. The order is rooted with members of Russulales and Gloeophyllales, and the family Corticiaceae is in a well-supported clade indicated with an arrow (Figure 2). Ten genera were recovered in the Corticiaceae, enclosed by light gray boxes: *Erythricium* J. Erikss. and Hjortstam*, Disporotrichum* Stalpers*, Marchandiomyces* Diederich and D. Hawksw., *Laetisaria* Burds., *Waitea* Warcup and P. H. B. Talbot*, Basidiodesertica* Maharachch., Wanas. and Al-Sadi*, Tretopileus* B. O. Dodge*, Giulia* Tassi*, Corticium*, and the new genus *Mycobernardia*. An identification key to distinguish these genera is provided in the following.

In the nLSU + ITS + SSU + mtSSU phylogram (Figure 2), the genus *Erythricium* appeared in a strongly supported clade (PP = 1.00) that comprised six species, including the isolates of the new species *Erythricium vernum*. “*Lawreymyces palicei*” is nested in this clade and is discussed below. *Marchandiomyces* formed a clade with full support and consists of five species, one of which is undescribed. *Laetisaria* was also recovered in a fully supported clade and sister to *Marchandiomyces*. The type material of *Laetisaria agaves* Burds. and Gilb. and *Corticium endoxylon* Duhem and H. Michel, both sequenced in this study, are included in the *Laetisaria* clade. The genus *Corticium* formed a fully supported clade sister to a clade containing the three small asexual genera *Basidiodesertica, Tretopileus*, and *Giulia*. Samples of *Galzinia incrustans* Parmasto found an isolated clade sister to the rest of Corticiaceae, and the new genus *Mycobernardia* is established for this taxon (below).

**Figure 3:**
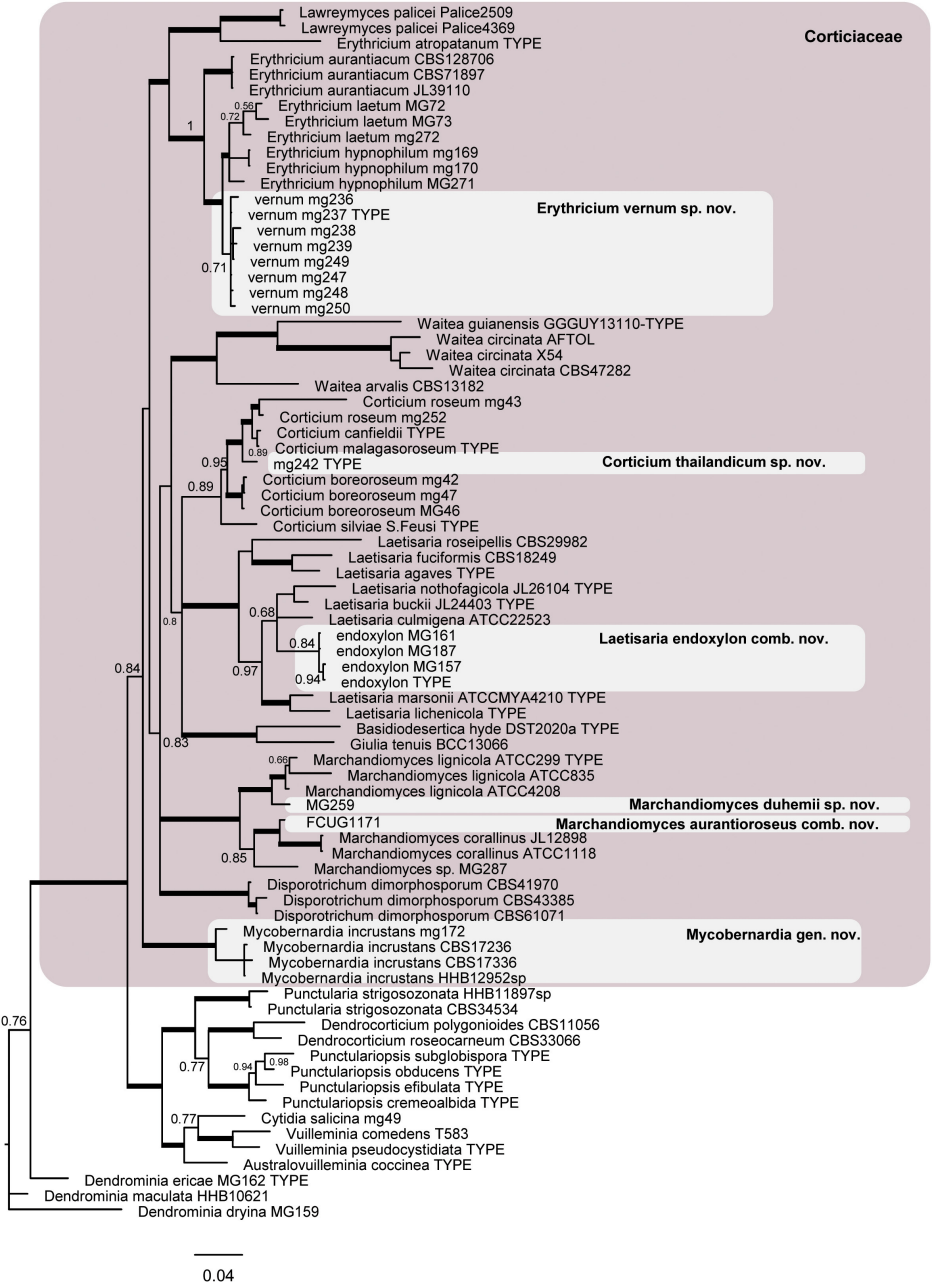
Phylogram of the nLSU-ITS dataset representing the Corticiaceae family (pink box) and taxonomic novelties in the Corticiaceae. Branches in bold have the posterior probability = 1.00. The scale bar represents the expected changes per site.

The phylogram obtained from the Bayesian analyses of dataset 2 (LSU + ITS) indicates the position of the taxonomic novelties introduced in this study within the Corticiaceae (Figure 3), including one new species in *Erythricium*, one new species in *Corticium*, and one new species in *Marchandiomyces*. The four isolates of *Corticium endoxylon*, including its type, are located in the *Laetisaria* clade; therefore, a new combination is proposed below. Isolates of *Galzinia incrustans* are accommodated in the new genus *Mycobernardia*.

**Figure 4:**
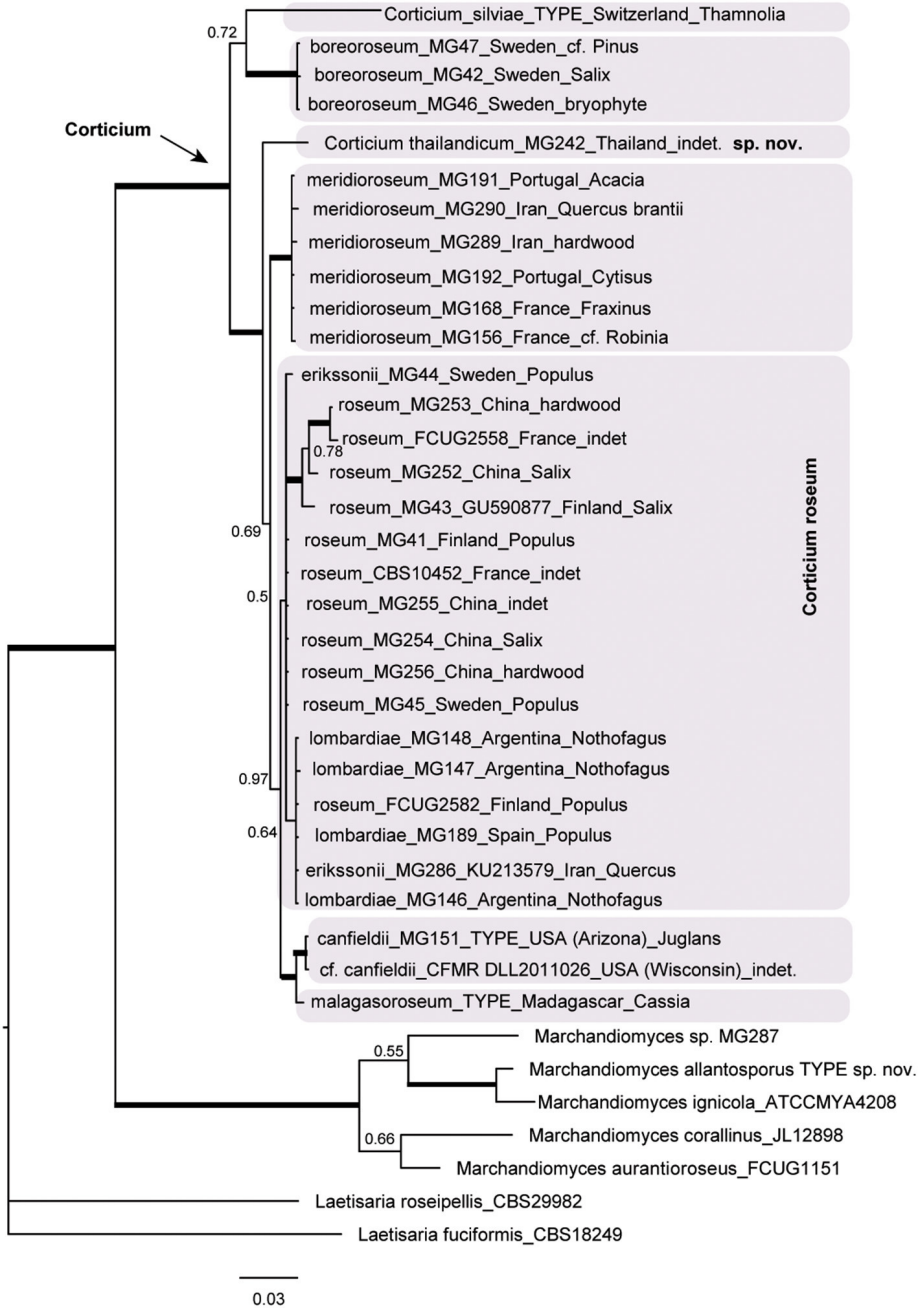
The ITS phylogram of species in *Corticium*. Each pink box represents a *Corticium* species. Country of collection and substrates are shown on the branches. Branches in bold the posterior probability = 1.00. The scale bar represents the expected changes per site.

**Figure 5:**
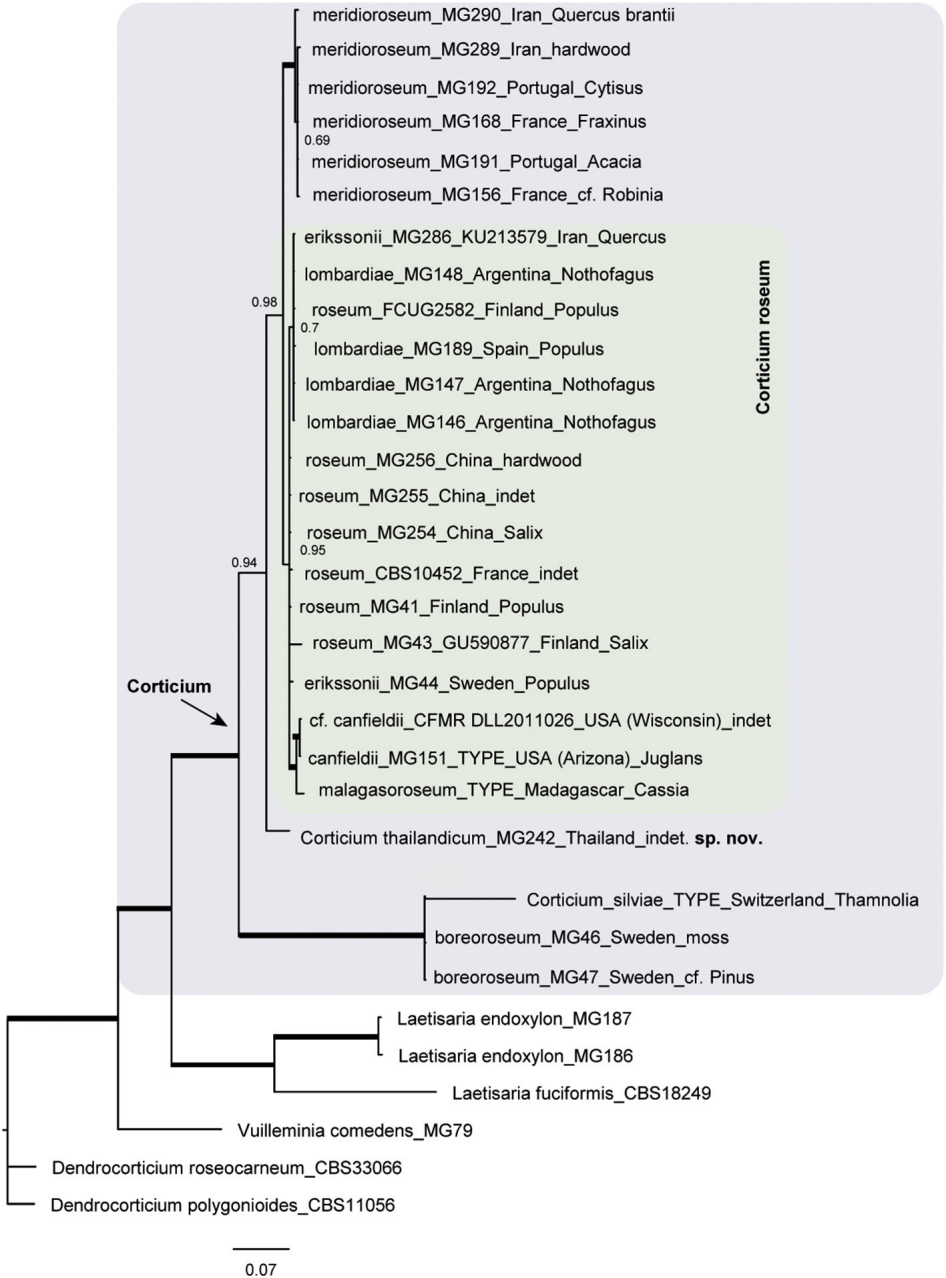
Phylogram of the combined IGS-ITS dataset for the *Corticium roseum* group. The pink box marks the *Corticium* clade, and the inside green box marks *C. roseum*. Country of collection and substrates are shown on the branches. Branches in bold have the posterior probability = 1.00. The scale bar represents the expected changes per site.

The resulting Bayesian phylogenetic trees for dataset 3 (ITS) and dataset 4 (ITS + IGS) are shown in Figures 4, 5, respectively. In the *Corticium* clade, the country and substrate are shown at each terminal. In both phylograms, *Corticium meridioroseum* Boidin and Lanq. and *C. boreoroseum* Boidin and Lanq. form distinct clades, while isolates of *C. erikssonii* Jülich*, C. lombardiae* (M. J. Larsen and Gilb.) Boidin and Lanq., and *C. roseum* cluster together. However, the two phylograms differ in the positioning of *C. canfieldii* (M. J. Larsen and Gilb.) Boidin and Lanq. and *C. malagasoroseum* Duhem: the ITS tree (Figure 4) shows them in separate clades, but in the ITS + IGS tree, they merge with *C. roseum* (Figure 5). Therefore, we propose to synonymize *C. erikssonii* and *C. lombardiae* under *C. roseum*, but to maintain *C. canfieldii* and *C. malagasoroseum* as separate species. Regarding the country and substrates, it is shown that *C. roseum* has a wide distribution range, in northern and western Europe, and east and west Asia—where it mainly grows on *Populus* and *Salix*—as well as southern South America. The distribution of *Corticium canfieldii* and *C*. *malagasoroseum* appears limited to the United States and Madagascar, respectively (The genus *Corticium* and the species in the *C. roseum* group are discussed in the following).

Regarding the nutritional character state reconstruction analyses, both the parsimony and the Bayesian approaches identified saprotrophy as the ancestral state in the Corticiaceae (Bayes factor test > 5 in the case of the Bayesian analysis).

### Taxonomy

***Mycobernardia*** Ghobad-Nejhad **gen. nov**.

Mycobank no.: MB839369.

*Diagnosis:* The genus is characterized by ceraceous, corticioid basidiomata, a monomitic hyphal system with clamps at all septa, subcylindrical to suburniform basidia with occasional internal repetition, and curved, allantoid basidiospores.

*Etymology:* myco + Bernard, in memory of Bernard Duhem (1964–2016), French mycologist at PC herbarium, Paris.

*Type species: Mycobernardia incrustans* (Parmasto) Ghobad-Nejhad **comb. nov**.

Mycobank no.: MB840807.

≡ *Galzinia incrustans* Parmasto, Eesti NSV Tead. Akad. Toim., Biol. seer 14(2): 225 (1965).

= *Corticium roseopallens* Burt, Proceedings of the Boston Society for Natural History 33: 173 (1907) (Type: PC!).

*Basidiomata* effused, thin, adnate, ceraceous; hymenial surface smooth, cream-colored with a faint rose tint. *Hyphal system* monomitic, all hyphae with clamps, richly branched. *Subiculum* thin. *Hymenium* dense, with abundant probasidia and basidia. *Basidia* subcylindrical to suburniform, occasionally with internal repetition. *Cystidia* and *dendrohyphidia* none. *Basidiospores* curved, allantoid, thin-walled, CB–, IKI–.

*Notes: Mycobernardia* is monotypic, and its type was previously assigned to *Galzinia* because of its curved, allantoid basidiospores and internally repetitive basidia. *Galzinia* species, including the generic type *G. pedicellata* Bourdot, develop very thin, almost invisible, watery gray basidiomata. In contrast, basidiomata in *Mycobernardia* are thicker, distinct, ceraceous, and cream-colored. Species in the two genera also differ in their nuclear behavior, subnormal in *G. pedicellata* and heterocytic in *B. incrustans* (Nobles, 1937; Stalpers, 1978; Boidin and Lanquetin, 1984). Molecular analyses of sequences from the type specimen of *G. longibasidia* Hallenb. place it in the Agaricales (Li et al., 2016).

**Figure 6:**
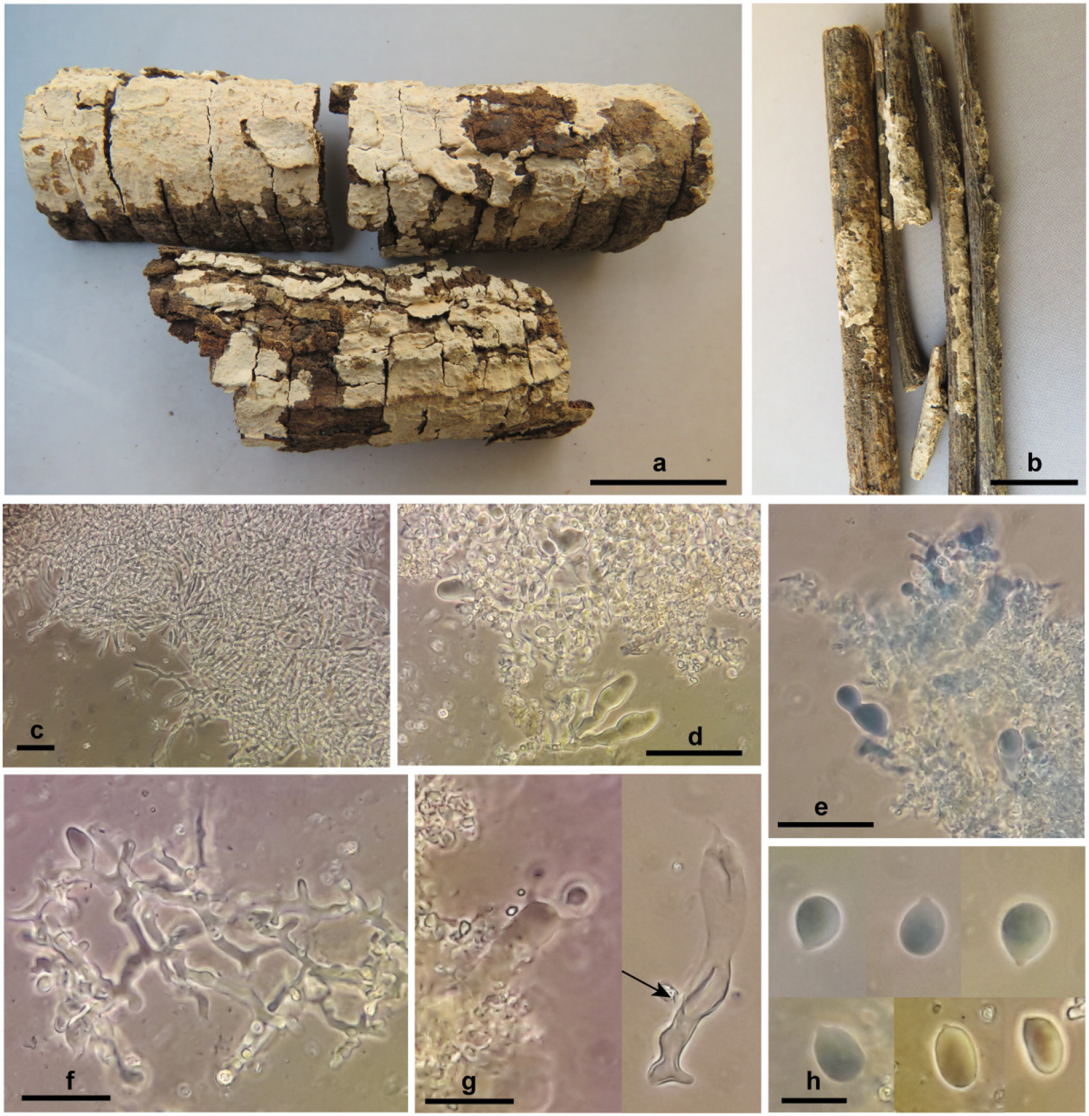
*Corticium thailandicum* sp. nov. **(a)** basidiomata, holotype; **(b)** basidiomata, paratype; **(c)** subicular hyphae; **(d–f)** hymenium with basidioles and dendrohyphidia; **(g)** basidium embedded in crystalized dendrohyphidia (left), an old basidium with wall thickening in lower part (right, arrow); **(h)** basidiospores. Scale bars **(a)** 2 cm; **(b)** 1 cm; **(c–e)** 50 μm; **(f,g)** 20 μm; **(h)** 10 μm. Photos: M. Ghobad-Nejhad.

***Corticium thailandicum*** Ghobad-Nejhad **sp. nov**. (Figure 6)

Mycobank no.: MB839371.

*Diagnosis:* The species is characterized by thin, light pinkish cream, ceraceous, and closely adnate basidiomata, broadly ellipsoid to subglobose basidiospores measuring 10–13 × (6.5) 7–9 μm, and by the occurrence in a dry dipterocarpous forest.

*TYPUS*: Thailand, Chiang Mai, Mae Rim District, Mae Raem, Queen Sirikit Botanical Garden, in dry deciduous forest with *Dipterocarpus obtusifolius, D. tuberculatus, Shorea obtusa*, and *Hopea odorata*, on corticated hardwood branches, 15.I.2012, Ghobad-Nejhad 3013 (ICH, holotype; RAMK, isotype).

*Etymology:* The species epithet refers to the country of collection.

*Basidiomata* effused, thin, closely adnate, confluent, tightly attached to the substrate, ceraceous; hymenial surface smooth, ca. 0.2 mm thick, light pinkish cream; context absent; margin distinct to slightly thinning out. *Hyphal system* monomitic, all hyphae with clamps, hyaline, smooth, CB–, IKI–. *Subiculum* narrow, loose, non-agglutinated, hyphae 2.7–3.7 μm diam., richly branched, with clamps at all septa, walls thin but distinct. *Subhymenium* narrow, dense, hyphae 3.7–5.5 μm diam., richly branched, with thin to thickened walls. *Hymenium* very dense, with abundant dendrohyphidia and numerous probasidia, mature basidia rare. *Basidia* at first as probasidia with various shapes, ovoid, subglobose, angular or prolonged, becoming clavate, irregularly constricted, flexuous, with percurrent proliferation, 33–55 × 7.5–12 μm, walls thickened especially at lower part, with clamps at base, contents refractive in KOH, bearing four stout sterigmata, 7 × 2 μm. *Cystidia* none. *Dendrohyphidia* abundant, richly branched, 1.5–3 μm diam., with clamps, with fine crystals in older parts of specimen. *Basidiospores* broadly ellipsoid to subglobose, 10–13 × (6.5) 7–9 μm, Q = 1.1–1.9, walls thin, smooth, CB–, IKI–.

*Paratype:* Same locality as type, on hardwood twigs, 15.I.2012, Ghobad-Nejhad 3012 (ICH, RAMK). Ex-paratype rDNA sequences: ITS (MW805868), nLSU (MW805831).

*Habitat and Distribution: Corticium thailandicum* is currently known from a dry deciduous dipterocarp forest in Chiang Mai, northern Thailand.

**Figure 7:**
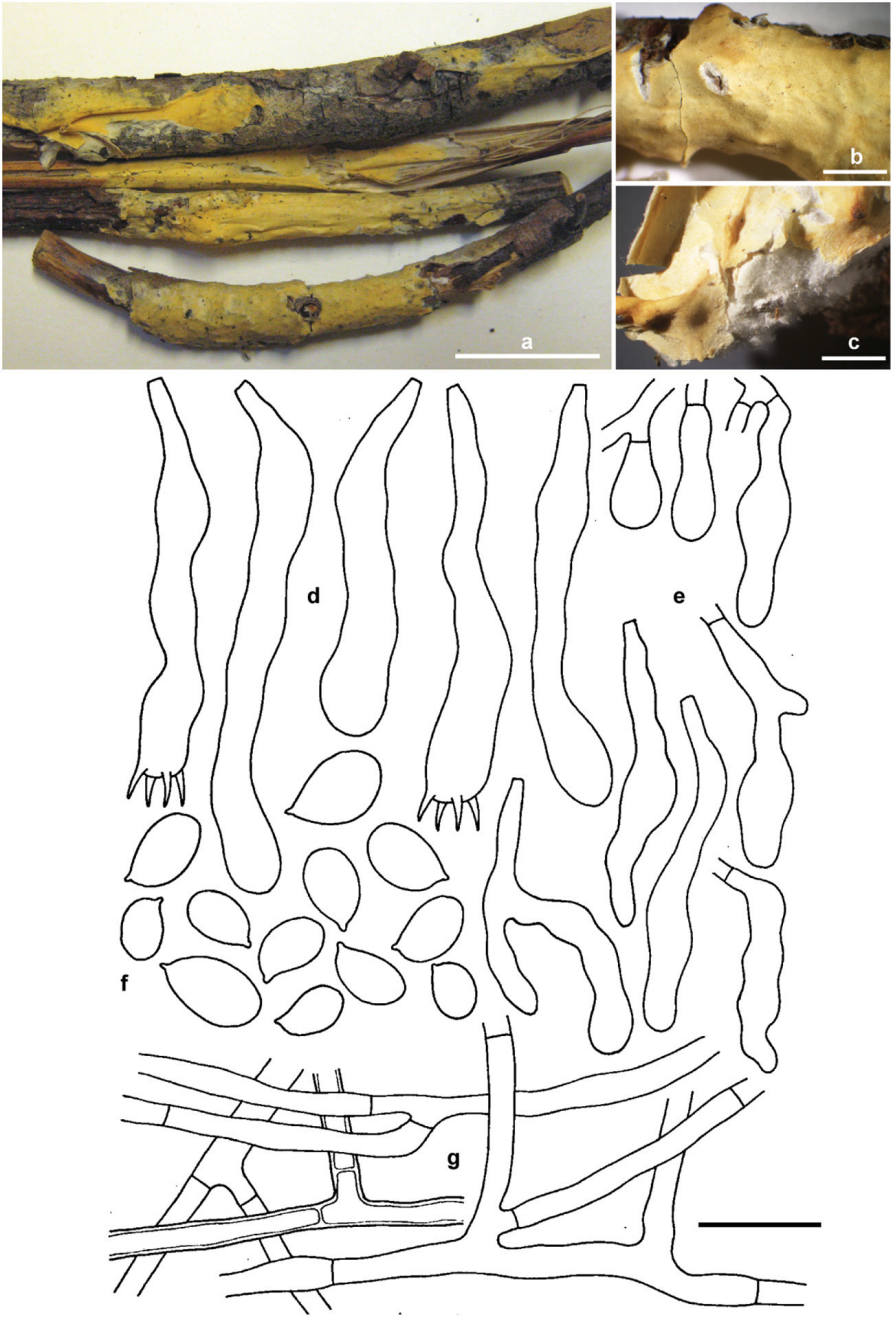
*Erythricium vernum* sp. nov. **(a)** basidiomata, holotype; **(b,c)** basidiomata, RLG-7886; **(d)** basidia; **(e)** basidioles; **(f)** basidiospores; **(g)** subicular hyphae. Scale bars **(a)** 2 cm; **(b,c)** 0.5 cm; **(d–g)** 20 μm. Photos **(a)** by M. Ghobad-Nejhad; **(b,c)** by K. Nakasone. Line drawing: reproduced with permission from Gilbertson (1973).

***Erythricium vernum*** Ghobad-Nejhad, Nakasone and Ginns **sp. nov**. (Figure 7).

Mycobank no.: MB839372.

*Diagnosis:* Differs from other *Erythicium* species by its reddish orange (when fresh), buff to light yellow (when dry), pellicular basidiomes, white, cottony context, thick-walled subicular hyphae, lack of short-celled, isodiametric hyphae, dense hymenium, acyanophilous basidiospore walls, and association with conifer litter.

*TYPUS*: USA, Montana, Flathead, Coram Expt. Forest, Flathead National Forest Hungry Horse, on conifer twigs and ninebark, 5.V.1975, M. J. Larsen s.n. (CFMR FP-133815, holotype; ICH, isotype). Ex-type rDNA sequences: ITS (MW805865), nLSU (MW805828).

*Etymology:* The species epithet refers to the fruiting time of the species in spring and frequent association with receding snow banks.

*Basidiomata* effused, adnate, confluent, loosely attached, pellicular; hymenial surface smooth with occasional folds, 250–750 μm thick, reddish orange when fresh, light yellow, light orange to grayish orange when dry, with some darker brownish orange areas; context white, cottony to subfelty; margin thinning out, white, fibrillose. *Hyphal system* monomitic, all hyphae simple-septate, hyaline, smooth, CB–, IKI–. *Subiculum* a loose, non-agglutinated, open tissue, hyphae 3.5–7.2 μm diam., moderately branched sometimes at right angles or forming H-connections, often septate near branch nodes, walls up to 4 μm thick. *Subhymenium* narrow, poorly defined, hyphae 3.5–5 μm diam., moderately branched, walls thin to thickened in 5% KOH. *Hymenium* up to 70 μm thick, a dense palisade of mostly immature and rare mature basidia, and few hyphidia. *Basidia* clavate, irregularly constricted, flexuous, 45–75 × 7–11 μm, simple-septate at base, stalked or with a bladder-like probasidia, with moderate percurrent proliferation, bearing four stout sterigmata, 7 × 2 μm. *Cystidia* none. *Hyphidia* scarce, filamentous, 20–35 × 1.5–2.2 μm. *Basidiospores* broadly ellipsoid, often flattened adaxially and slightly fusoid, with a distinct apiculus (9.3–) 9.7–12 (−12.8) × 5.7–7.2 (−8) μm, Q = 1.6–1.7, walls thin to slightly thickened, smooth, CB–, IKI–, occasionally germinating from apiculus or from lateral wall, contents more or less refractive in KOH.

*Paratypes:* Canada, British Columbia, Osoyoos, Mt Kobol road, 15.8 km from junction with Highway 3, on upper surface of old *Pseudotsuga menziesii* cone on ground near receding snow bank, 12.V.2007, leg. O. Ceska, det. J. Ginns 11684 (DAOM). USA, Arizona, Treasure Park, Pinalenio Mts., Coronado National Forest, Graham County, on twigs, leaves, and corticated wood of *Pseudotsuga menziesii*, 16.VI.1968, R.L. Gilbertson (RLG-7886, CFMR). USA, Arizona, Pima Co., Santa Catalina Mts., Summerhaven, on bark of 1 cm diam. *Pseudotsuga menziesii* twig on ground, 8.V.1970, R.L. Gilbertson 9356 (DAOM 172906). USA, Montana, Flathead, Coram Expt. Forest, Flathead National Forest Hungry Horse, on conifer branches, 30.V.1974, M.J. Larsen (FP-133814, CFMR), on conifer and ninebark twigs, M.J. Larsen (FP-133817, CFMR). USA, Washington, Chelan Co., Wenatchee National Forest, Lake Wenatchee area, Spencer Creek, off Tieton Road, on fallen *Pseudotsuga menziesii* twigs, *Pinus ponderosa* needles and dead grass near receding snow banks, 8.V. 1999, J. Ginns (10782), J. Lindgren and M. Rogers (DAOM 226340). USA, Washington, Yakima Co., Mt St Helens, E side of White Pass off Highway 12, on overwintered *Pseudotsuga menziesii* cone on ground near receding snow bank, 15.V.1993, CISPUS foray, communicated by S. A. Redhead, det. J. Ginns 11912 (DAOM).

*Habitat and Distribution: Erythricium vernum* is associated with conifer litter and wood, especially *Pseudotsuga menziesii* and receding snow banks. It is currently known from western USA (Arizona, Montana, and Washington) and Canada (British Columbia).

*Notes:* The specimens of *E. vernum* (RLG 7886, 7883, 9356, 9393, and 7839) were misidentified by Gilbertson (1973) as *Corticium lepidum* (Romell) Bourdot and Galzin, and Eriksson et al. (1978) suggested that it belongs to *Erythricium*. Later, Ginns and Lefebvre (1993) called this taxon “*Erythricium* species A.”

Some basidiospores in *E. vernum* were very large, up to 17 × 10 μm. Based on our experience, larger than normal-sized basidiospores are not uncommon in the Corticiales, such as in species of *Corticium* and *Vuilleminia* Maire.

**Figure 8:**
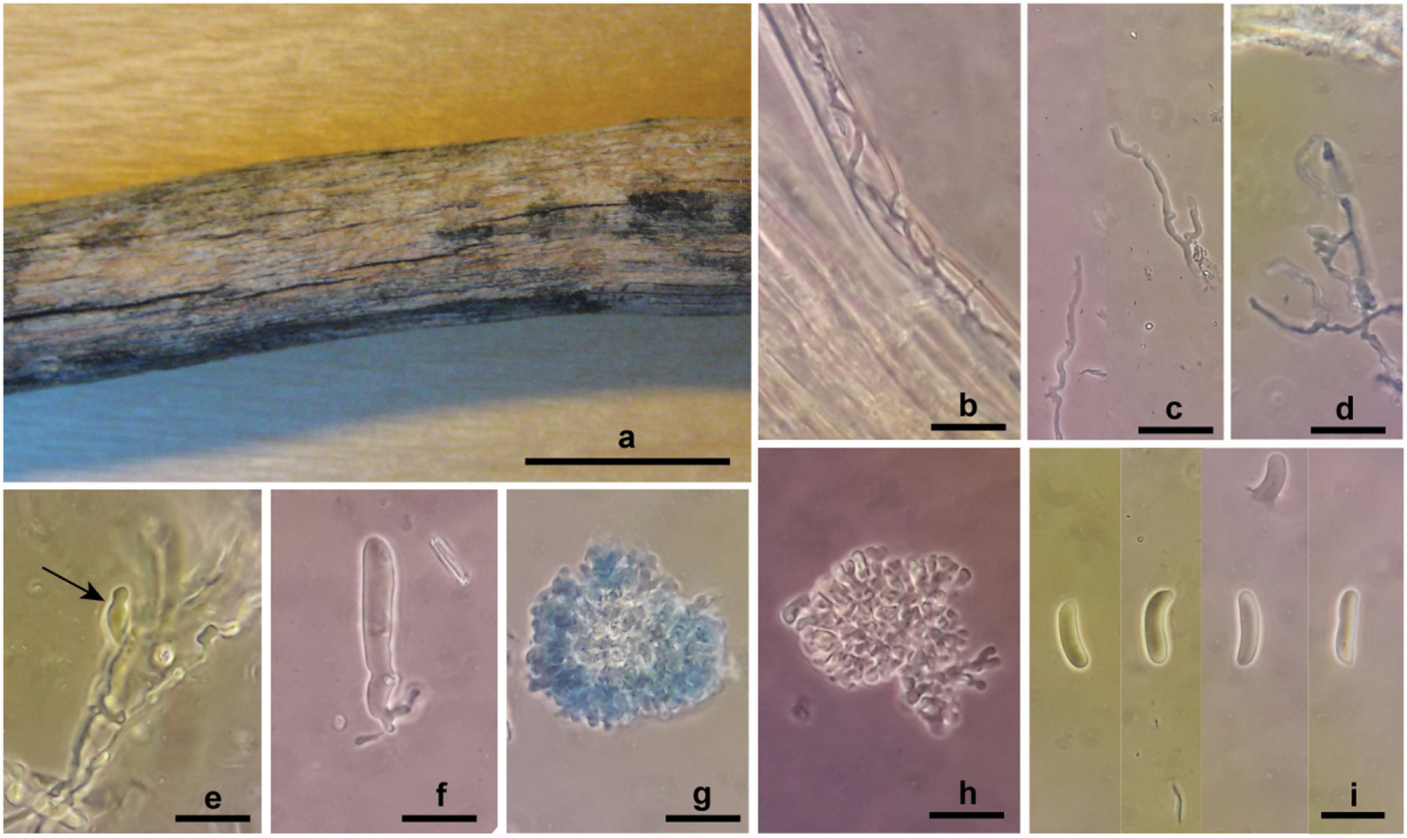
*Marchandiomyces allantosporus* sp. nov. from holotype. **(a)** Basidiomata; **(b)** hyphae in wood vessel; **(c,d)** hyphae; **(e)** basidiole (arrow); **(f)** basidium; **(g,h)** hyphelium; **(i)** basidiospores. Scale bars, **(a)** 1 cm; **(b–i)** 20 μm.

***Marchandiomyces allantosporus*** Ghobad-Nejhad **sp. nov** (Figure 8).

Mycobank no.: MB839373.

*Diagnosis:* The species is characterized by its endoxylon growth habit embedded in decorticated conifer wood, and large allantoid basidiospores (12.5) 15.5–25.5 × 4.5–6.5 (8) μm.

*TYPUS*: France, Var, Montauroux, [Bois Communal du] Défens, on decorticated branch of *Juniperus* cf. *communis*, 2.X.2002, leg. H. Michel, herb. B. Duhem 5354 (PC 0085935, holotype; ICH, isotype). Ex-type rDNA sequences: ITS (MW805877).

*Etymology:* The species epithet refers to the allantoid basidiospores.

*Basidiomata* almost invisible, detectable as wood discoloration with a rose tint, with structures embedded inside wood. *Hyphal system* monomitic, hyphae infrequent, with clamps, hyaline, smooth, CB–, IKI–. *Subiculum* none. *Subhymenium* very narrow, loose, hyphae 2.5–3.7 μm diam., moderately branched, walls thin. *Basidia* sparsely formed, at first as ovoid probasidia, becoming clavate, irregularly constricted, flexuous, 40–55 × 6.5–10 μm, walls thin to moderately thickened, with clamps at the base, with four stout sterigmata. *Cystidia* and *dendrohyphidia* none. *Hyphelia* occasionally present, spherical to subspherical, 37–75 × 28–60 μm, consisting of densely packed short-celled, radiating hyphae, branches bifurcate at the tips, hyphae 3–5 μm diam. *Basidiospores* allantoid (12.5) 15.5–25.5 × 4.5–6.5 (8) μm, Q = 2–4.2, walls thin, smooth, CB–, IKI–.

*Paratypes*: France, Var, Montauroux, [Bois Communal du] Défens, on decorticated branch of *Pinus* sp., 3.XII.2001, leg. H. Michel, Duhem 5352 (PC 0085933, ICH); on decorticated branch of *Pinus halepensis* or *Juniperus*, 2.X.2002, leg. H. Michel, herb. B. Duhem 5353 (PC 0085934, ICH).

*Habitat and Distribution:* The species is currently known from France, growing on decorticated conifer branches (*Juniperus, Pinus*).

*Notes:* The hyphelia are seen as small hyphal aggregates (ca. 50 μm diam. in average) under the microscope. These asexual structures were illustrated by Eriksson and Ryvarden (1976, p. 784) for *Laeticorticium pulverulentum* (Erikss. and Ryv.) Erikss. and Ryv. (syn. *Corticium erikssonii*), supposed to function as diaspore for dispersal. The same structures, called “sclerotia,” have been shown for some bulbil-forming Corticiaceae members, e.g., by DePriest et al. (2005) for *M. lignicola* Lawrey and Diederich. Duhem (2011) provided notes and illustrations on specimens of this species, with an attempt to identify them at genus level.

## Discussion

In this study, we examined the modern delimitation of the family Corticiaceae and its genera with the aid of combined molecular data. This is the first comprehensive account of phylogenetic relationships of taxa in this long-used family name. We summarize the common features of Corticiaceae as the following:

- Basidiomata (if present) in simple corticioid (crust) form
- Prevalence of pink coloration in basidiomata/colonies and in spore print
- Monomitic hyphal system
- Lack of amyloidy-dextrinoidy reaction
- Generally large basidia and basidiospores
- Lack of cystidia
- White rot (in case of wood-inhabiting taxa).

According to our results, Corticiaceae currently contains ten monophyletic genera, with saprotrophic, parasitic, and lichenicolous species, besides an endolichenic taxon. The genera *Corticium, Erythricium, Laetisaria*, and *Marchandiomyces* encompass more than one type of nutritional mode. The analyses of character state reconstruction strongly suggested saprotrophy as the ancestral mode of nutrition in this family, while parasitism and lichenicolous habits are the results of more recent transitions. Based on phylogeny, DePriest et al. (2005) and Lawrey et al. (2008) had speculated the lignicolous habit to be an ancestral feature in *Marchandiomyces*, while our evolutionary analyses clearly show this for the entire family. Parasitic taxa of the Corticiaceae were recently discussed by Jayawardena et al. (2019), and lichenicolous taxa are discussed in the following.

In Figure 2, all genera are represented by their type species, except for *Tretopileus* the type of which (*T. opuntiae* B. O. Dodge) has not been sequenced yet. The monotypic genera in the Corticiaceae include *Basidiodesertica, Mycobernardia, Disporotrichum*, and *Giulia*.

We also scrutinized available data on the number of nuclei per basidiospore, mating type, and nuclear behavior in the family Corticiaceae when known (Supplementary Table 2, see also Jackson, 1950; Eriksson, 1958; Hallenberg, 1986; Boidin and Lanquetin, 1995, 1997). Among the 12 species for which such data are available, most species have binucleate basidiospores. The mating type in most of the species in bipolar. The nuclear behavior varies among species.

Identification keys are provided below to the genera in Corticiaceae, and to the species in *Corticium, Erythricium, Laetisaria*, and *Marchandiomyces*. A key to the species of *Waitea* is available in Gruhn and Ghobad-Nejhad (2021).

Key to the genera in Corticiaceae^*^

1. Basidiomata essentially corticioid (see also bulbil-forming species in *Marchandiomyces* and *Laetisaria*)………………2
1. Basidiomata absent, only asexual morph present (see also bulbil-forming species in *Marchandiomyces* and *Laetisaria*)………………………………………………7
2. Basidia and basidioles with conspicuous wall thickening especially basally, always with dendrohyphidia and clamps…………………………………………***Corticium***
2. Basidia and basidioles not as above, dendrohyphidia and clamps present or absent…………………………………3
3. Basidia subcylindrical to urniform………………………‥ 4
3. Basidia clavate to elongated………………………………. 5
4. Clamps present, thick-walled basal hyphae and short-celled subhymenial hyphae absent, basidia occasionally with internal repetition, some cystidioles may be present, basidiospores allantoid…………………***Mycobernardia*** gen. nov.
4. Clamps absent, thick-walled basal hyphae and short-celled subhymenial hyphae present, basidia without internal repetition, cystidioles absent, basidiospores ellipsoid…………………………………………***Waitea***
5. Colonies with different shades of red to pink colors, clamps present or absent, bulbils (if present) pink to red, basidiospores ellipsoid to ovoid or allantoid………………………………………………6
5. Colonies with different shades of pink, orange and buff colors, bulbils (if present) orange, clamps totally lacking, basidiospores ellipsoid-fusoid, often flattened adaxially…………………………‥ ***Erythricium***
6. Bulbils (if present) coral red, basidiomata-producing species as tiny patches around lenticel or almost invisible inside wood, with a rose tint, without dendrohyphidia, basidiospores up to 20–25 μm long, basidia with 4 sterigmata………………………………***Marchandiomyces***
6. Bulbils (if present) pink, basidiomata-producing species crustose to membranaceous, usually with dendrohyphidia, basidiospores <20 μm long, basidia with 2–4 sterigmata…………………………………***Laetisaria***
7. Asexual morph coelomycetous, pycnidial, conidia appendaged…………………………………………***Giulia***
7. Asexual morph hyphomycetous…………………………8
8. With synnemata, verticillate clamps absent………………. 9
8. Without synnemata, conidia dimorphic, some hyphae bearing verticillate clamps…………………………***Disporotrichum***
9. Synnemata cylindrical to subulate, with a pink stroma at base………………………………………***Basidiodesertica***
9. Synnemata capitate, stromatic tissue absent…………………***Tretopileus*** (only *T. sphaerophorus*)

^*^This key is preliminary and may not satisfactorily identify some genera especially those with bulbil-forming species.


### Corticium

*Corticium* is the type genus of the family Corticiaceae and has a checkered past as a dumping ground for basidiomycetes with simple corticioid basidiomata (Donk, 1963). With the emergence of molecular data, the genus has become more narrowly defined as many species were transferred to other genera, families, and orders. Studies by Boidin et al. (1968), Boidin and Lanquetin (1984), Boidin and Gilles (1998) and later by Duhem and Michel (2006, 2009) contributed to the definition of *Corticium* in its strict sense, largely corresponding to the concept of *Laeticorticium* Donk in the studies by M. J. Larsen (Larsen and Gilbertson, 1974, 1978; Larsen and Nakasone, 1984), a superfluous synonym of *Corticium*. The present study confirms that *Corticium* is a monophyletic genus for species with conspicuous wall thickening at the bases of basidia and basidioles, flexuous basidia developing from bladder-like basidioles, and always bearing dendrohyphidia and clamped hyphae.

We recognize 14 species in *Corticium* (see the following key), of which seven species are confirmed phylogenetically. The basidiomata color is pink in most of the species, but there are also species with whitish to grayish buff colors. Only *C. silviae* Diederich, E. Zimm. and Lawrey is lichenicolous, while the remainder are saprotrophic.

Key to the known species of *Corticium* (updated from Duhem and Michel, 2009)

1. Basidiomata lichenicolous………………‥………***C. silviae***
1. Basidiomata saprotrophic on wood, bryophyte or litter…………….………………………………………2
2. On conifers, in North or South America…………………‥3
2. Mostly on hardwoods in different continents………………6
3. Dendrohyphidia golden brown, known on *Pinus* cone from Mexico………***C***. ***durangense*** (M. J. Larsen and Gilb.) Boidin and Lanq.
3. Dendrohyphidia colorless…………………‥……………4
4. Basidiomata thin, grayish white, on bark of *Pseudotsuga*, basidiospores cylindrical to narrowly ellipsoid, very large, l9–23 × 8–10 μm, dendrohyphidia scarcely developed………………***C. griseoeffusum*** (M. J. Larsen and Gilb.) Ginns and M. N. L. Lefebvre
4. Basidiomata pink at least when fresh, basidiospores and dendrohyphidia different………………‥………………5
5. Basidiomata pinkish buff when fresh, whitish cream when dry, with small sclerotia, hyphae dissolving in KOH, basidiospores 12–15 × 9–11 μm, on various conifers………………***C. minnsiae*** (H. S. Jacks.) Boidin and Lanq.
5. Basidiomata rose to pink, basidiospores 9–11 (13) × 5–7.5 (9) μm, on *Pinus*………………***C. pini*** (H. S. Jacks.) Boidin and Lanq.
6. Basidiomata with various shades of pink color……………7
6. Basidiomata cream to gray, devoid of pink coloration……………………….……………………12
7. With rhizomorphs (basidiomata starting as thin rhizomorphs then thickening into fibrillose margin), basidiospores 6–10 × 4–5 μm, on various substrata such as wood, debris, and bryophytes, mostly in northern Europe……………………………………***C. boreoroseum***
7. Without rhizomorphs, basidiospores larger………………8
8. Basidiospores (9) 11–13 (14) × 7–9 μm, distribution in Mediterranean areas……………………***C. meridioroseum***
8. Basidiospores and distribution otherwise…………………9
9. Basidiospores 7–9 × 5.5–6.6 μm, known from Madagascar, on *Cassia ………………………………*.***C. malagasoroseum***
9. Basidiospores larger, known from other parts of the world……………………………….…………………10
10. Basidiomata thin, ceraceous, closely adnate, basidiospores 10–13 × 7–9 μm, known from Thailand………***C. thailandicum*** sp. nov.
10. Basidiomata soft felty to subfelty, known from North America or worldwide………………………………………‥…11
11. Basidiomata surface cracked, basidiospores 13–15 × 6–8.5 μm, known from North America, on *Juglans………………………………………**C. canfieldii***
11. Basidiomata surface not cracked, may form large patches, basidiospores 10–24 × (5) 8–12 μm, common species and widely distributed, on various hosts, in the Northern Hemisphere especially on *Salix* and *Populus………**C. roseum*** (incl. *C. lombardiae* and *C. erikssonii*)
12. Dendrohyphidia tips collapsed or capitulate and cystidia-like, basidiomata yellowish white to grayish yellow, on *Vitis* and *Nyssa* in USA, basidiospores 12–15 (18) × 7–8 μm………………***C. appalachiense*** (Burds. and M. J. Larsen) M. J. Larsen
12. Dendrohyphidia tips not collapsed and not capitulate, basidiomata very thin and pale cream to buff………‥‥… 13
13. Basidiomata confluent, basidiospores 12–14 × 6.5–8 μm, known from USA on *Myrica*………***C. floridense*** (M. J. Larsen and Nakasone) M. J. Larsen
13. Basidiomata beginning from minuscule initials, basidiospores 15–18 × 8–11 μm, known from USA on various hosts (incl. *Sabal palmetto, Quercus, Morus, Cornus, Juglans*)………………………***C. mississippiense*** (Lentz) M.J. Larsen

### The *Corticium roseum* Group

The type species of the genus *Corticium* is *C. roseum*, which is known to have some resembling counterparts differing mainly in basidiospore sizes: *C. erikssonii, C. lombardiae, C. meridioroseum, C. boreoroseum*, and also two species each known from a single collection, viz. *C. malagasoroseum* described from Madagascar and *C. canfieldii* from Arizona, USA.

Here we examined the phylogenetic relationships among taxa in the *Corticium roseum* group by analyses of ITS and IGS sequence data. The IGS region is one of the rapidly evolving portions of the nrDNA and often distinguishes fungal taxa at the species level (White et al., 1990; O'Donnell et al., 2009; Wurzbacher et al., 2019). However, according to the published studies, its discriminatory power is variable. In the study by Coetzee et al. (2018), IGS was only partially useful to discriminate among *Armillaria* (Fr.) Staude species. In the case for *Macowanites messapicoides* Llistos. and J. M. Vidal (sequestrate-hypogeous) and *Russula messapica* Sarnari (agaricoid-epigeous), identical IGS-RFLP patterns allowed Martín et al. (1999) to confirm that the two taxa were different morphotypes of the same species, *R. messapica*. On the other hand, in a study related to *Hyphoderma macaronesicum* Tellería, M. Dueñas, Beltrán-Tej., Rodr.-Armas and M. P. Martín (corticioid Basidiomycota), the IGS region allowed to confirm the presence of a cryptic species, *H. paramacaronesicum* Tellería, M. Dueñas, J. Fernández-López and M. P. Martín (Martín et al., 2018).

In our study, the IGS + ITS sequence analyses confirmed the distinctiveness of *C. boreoroseum* and *C. meridioroseum*. *Corticium boreoroseum* is characterized by rhizomorphs, by having relatively small basidiospores (measuring 6–10 × 4–5 μm), and by its distribution in boreal regions mainly in northern Europe. Its basidiomata begin as thin rhizomorphs which become thicker upon development, leading to fibrillose margin. The species grows on various substrata such as wood, debris, and bryophytes. *Corticium meridioroseum* has a southern distribution in Mediterranean areas (also in Iran), growing on different hardwood plants, and its basidiospores measure (9) 11–13 (14) × 7–9 μm.

The IGS + ITS phylogram also suggested the synonymy of *C. erikssonii* and *C. lombardiae* with *C. roseum*, which was congruent with the ITS data. *Corticium erikssonii* (syn. *L. pulverulentum*) has been separated from *C. roseum* by producing asexual organs (hyphelia), whereas *C. lombardiae* differed by basidiospore size, and apparently different mating types (Larsen and Gilbertson, 1978; Boidin and Lanquetin, 1984). Specimens of *C. roseum* from Patagonia (Argentina) were formerly assigned to *C. lombardiae* (Greslebin and Rajchenberg, 2003) growing on *Nothofagus pumilio*. Our molecular analyses show that neither hyphelia nor the mating system are good characters for separation of *C. erikssonii* and *C. lombardiae* from *C. roseum*.

Unlike the ITS phylogram that suggests a distinctiveness of *C. malagasoroseum* and *C. canfieldii*, IGS data placed the two species in the *C. roseum* clade. We conclude *C. malagasoroseum* and *C. canfieldii* as two distinct species until more sequence data become available from these two, uncommon species. *Corticium roseum* is therefore shown to be a widely distributed species occurring in both hemispheres, with a wide ranging basidiospore size [10–24 × (5) 8–12 μm]. It has a preference for hardwood, especially *Populus* and *Salix* in the northern hemisphere.

### Erythricium

*Erythricium* was introduced by Eriksson and Hjortstam (1970) and is characterized by resupinate basidiomata with different shades of pink, orange, and buff colors (one species with orange bulbils), total lack of clamps, subhymenial hyphae with short cells, and relatively large basidiospores with firm walls. Basidiospores are broadly ellipsoid, often flattened adaxially and become fusoid. In our study, *Erythricium* is retrieved with six species with parasitic [*E. salmonicolor* (Berk. and Broome) Burds.], saprotrophic [*E. atropatanum* Ghobad-Nejhad and Hallenb., *E. laetum* (P. Karst.) J. Erikss. and Hjortstam*, E. hypnophilum* (P. Karst.) J. Erikss. and Hjortstam, *E. vernum*], and lichenicolous members [*E. aurantiacum* (Lasch) D. Hawksw. and A. Henrici]. The endolichenic “*Lawreymyces palicei*” nests in the *Erythricium* clade, but its formal combination into the latter genus is precluded as the name “*Lawreymyces*” was not validly published (see section Discussion below).

*Erythricium* species inhabit a wide range of substrata (bryophytes, lichens, dicotyledonous herbs, plant debris, and some fruit trees) and thrive in diverse habitats (Ghobad-Nejhad and Hallenberg, 2011). The most significant species is perhaps the plant pathogen *E. salmonicolor* (syn. *Necator decretus* Massee), which is the agent of “pink disease” in different plantations such as citrus, eucalypt, rubber, cacao, coffee, as well as many native plant trees in different parts of the world. Originally described from the Paleotropics, *E. salmonicolor* was soon detected in several cool temperate areas of northern hemisphere and appears to be spreading worldwide (Jayawardena et al., 2019).

*Erythricium chaparralum* Burds. and Gilb. described by Burdsall and Gilbertson (1982) is only known from the holotype that was studied here. It has ceraceous to subceraceous, grayish-orange to brown basidiomata firmly attached to the decayed, decorticated wood surface. Its tight and almost gelatinized texture clearly deviates from the *Erythricium* concept, and we believe it must be excluded from the genus. Herein, we propose a combination in the genus *Phanerochaete* P. Karst.

*Phanerochaete chaparrala* (Burds. and Gilb.) Nakasone and Ghobad-Nejhad **comb. nov**.

MycoBank no.: MB839374.

≡ *Erythricium chaparralum* Burds. and Gilb., Mycotaxon 15: 335 (1982) (Holotype: CFMR!)

Key to the known species of *Erythricium*

1. Colonies made of small, orange bulbils, rarely accompanied by basidiomata…………………………***E. aurantiacum***
1. Bulbils absent, basidiomata always present………………‥2
2. Basidiomata parasitic, walls of basal hyphae conspicuously thickened in KOH…………………………***E. salmonicolor***
2. Basidiomata saprotrophic, walls of basal hyphae not conspicuously changed in KOH………………‥…………3
3. On bryophytes, litter, and wood in coniferous forests……………………………………………………4
3. On deciduous wood or dicotyledonous herbaceous plants……………………………………………………5
4. Basidiospores 4.5–6 μm wide, mainly on bryophytes or conifer litter in northern Europe…………………***E. hypnophilum***
4. Basidiospores up to 8 μm wide, mainly on *Pseudotsuga* in North America…………………………***E. vernum*** sp. nov.
5. Basidiomata pellicular, subiculum composed of short-celled, wide hyphae, known from Europe and North America…………………………………………***E. laetum***
5. Basidiomata ceraceous, subiculum lacking, known from Iran………………………………………***E. atropatanum***

### Laetisaria

The genus *Laetisaria* is characterized by crustose to membranaceous basidiomata, the species usually having dendrohyphidia, basidiospores shorter than 20 μm, and basidia with 2–4 sterigmata. Three species are parasitic on grass leaves [*L. culmigena* (R. K. Webster and D. A. Reid) Diederich, Lawrey and Ghobad-Nejhad*, L. fuciformis* (Berk.) Burds., and *L. roseipellis* (Stalpers and Loer.) Diederich, Lawrey and Ghobad-Nejhad], while *L. agaves* grows on *Agave* leaves. With the inclusion of some bulbil-forming species, the concept of the genus was recently emended by Diederich et al. (2018); these include two lichenicolous [*L. lichenicola* Diederich, Lawrey and Van den Broeck and *L. buckii* (Diederich and Lawrey) Diederich, Lawrey and Ghobad-Nejhad], one lignicolous [*L. nothofagicola* (Diederich and Lawrey) Diederich, Lawrey and Ghobad-Nejhad], and one foliicolous species [*L. marsonii* (Diederich and Lawrey) Diederich, Lawrey and Ghobad-Nejhad]. Herein, we show that the genus concept is further extended to include also the wood-inhabiting *Corticium endoxylon*.

*Corticium endoxylon* is a curious species described by Duhem and Michel (2008) from France. It grows inside decorticated hardwood branches, leaving the growth area as bleached wood sometimes with a faint pink tint. We obtained sequences from the type and additional authentic material of *C. endoxylon*. Our phylogenetic analyses (Figures 2–4) show that the species clusters inside the genus *Laetisaria*. *Corticium lignigenum* Duhem and H. Michel described by Duhem and Michel (2006) has morphological characters very similar to *C. endoxylon*, differing only by smaller basidiospores. Therefore, the following new combinations are proposed.

*Laetisaria endoxylon* (Duhem and H. Michel) Ghobad-Nejhad **comb. nov**.

MycoBank no.: MB839058.

≡ *Corticium endoxylon* Duhem and H. Michel, Cryptog. Mycol. 29 (2): 114 (2008) (Holotype: PC!).

*Laetisaria lignigena* (Duhem and H. Michel) Ghobad-Nejhad **comb. nov**.

MycoBank no.: MB839375.

≡ *Corticium lignigenum* Duhem and H. Michel, Bull. Soc. mycol. Fr. 122(2-3): 146 (2007) [2006] (Holotype: PC!).

Regarding the evolution of nutritional modes in *Laetisaria*, as shown in Figure 2, the estimated character state in the node for *Laetisaria* clade is parasitism, preceded by a saprotrophic character state. Therefore, it appears that saprotrophy was lost and regained in the wood-dwelling species of *Laetisaria*.

Key to the known species of *Laetisaria*

1. Colonies made of minute pink bulbils, basidiomata unknown…………………………………………….…2
1. Basidiomata corticioid, bulbils unknown……………….…4
2. Bulbils foliicolous, on leaves of *Pandanus………**L. marsonii***
2. Bulbils on lichen thalli or on wood…………………….…3
3. Bulbils on wood of *Nothofagus……………**L. nothofagicola***
3. Bulbils lichenicolous, rarely on wood………………***L. buckii***
4. Basidiomata lichenicolous……………………***L. lichenicola***
4. Basidiomata on wood, grass, or *Agave*………………….…5
5. Basidiomata not visible, structures embedded inside the decorticated wood, detectable only as discolored or faint pink area……………………………………………‥………6
5. Basidiomata visible on grass or *Agave*…………………‥…7
6. Basidiospores 12–15 (−16.5) × 7–10 (−12) μm…………………………………………***L. endoxylon***
6. Basidiospores smaller, 8–12 × 4.5–6 μm…………………………………………***L. lignigena***
7. On *Agave* leaves…………………………………***L. agaves***
7. On grass leaves, parasitic…………………………………8
8. Clamps totally absent…………………………***L. fuciformis***
8. Clamps present at least on some septa……………………9
9. Basidia with four sterigmata, basidiospores 9–12 × 5–6 μm……………‥…………………………***L. roseipellis***
9. Basidia with two sterigmata, basidiospores 13–16 × 7–9.5 μm…………‥….,°………………………***L. culmigena***

### Marchandiomyces

*Marchandiomyces* is known to encompass bulbil-forming, lichenicolous, and lignicolous species (Diederich, 1990; DePriest et al., 2005; Diederich and Lawrey, 2007). The corticioid species *Corticium quercicola* was shown by Ghobad-Nejhad et al. (2010, as *Marchandiopsis quercina*) to be closely related to *Marchandiomyces*, and was eventually transferred to the genus by Hawksworth and Henrici (2015). Recently several species were excluded from *Marchandiomyces* by Diederich et al. (2018) in favor of *Laetisaria*. Currently, *Marchandiomyces* includes four saprotrophic lignicolous species, and one lichenicolous species (a *Marchandiomyces* sp. isolate MG287, found on *Quercus brantii* in Iran, is not fully characterized yet). The basidiomata-producing species generally form tiny patches around lenticels or are almost invisible inside wood, with a rose tint, without dendrohyphidia, and with large basidiospores.

One of the specimens of *Corticium quercicola* examined by us at herbarium S (specimen no. F119141) contained a note by Åke Strid who questioned if *C. quercicola* was synonymous with *Corticium aurantioroseum* P. Karst. We examined the type material of *C. aurantioroseum* deposited at herbarium H, and we confirm that they are conspecific. Therefore, the latter, older name takes the priority and a new combination is proposed below.

*Marchandiomyces aurantioroseus* (P. Karst.) Ghobad-Nejhad **comb. nov**.

MycoBank no.: MB839376.

≡ *Corticium aurantioroseum* P. Karst., Kritisk Öfversigt af Finlands Basidsvampar (11): 30 (1898) (Holotype: H!)
= *Corticium quercicola* Jülich, International Journal of Mycology and Lichenology 1 (1): 31 (1982)
= *Laeticorticium quercinum* J. Erikss. and Ryvarden, The Corticiaceae of North Europe 4: 777 (1976) (Holotype: GB!)
= *Marchandiomyces quercinus* **(**J. Erikss. and Ryvarden) D. Hawksw. and A. Henrici, Field Mycology 16 (1): 17 (2015)
= *Marchandiopsis quercina* (J. Erikss. and Ryvarden) Ghobad-Nejhad, Taxon 59 (5): 1530 (2010)

In this study, we targeted available DNA sequences of *Marchandiomyces* in GenBank, and we used most of them in our phylogenetic analyses. We also confirm that the ITS sequences with GenBank accession numbers LC462852 (isolate Kojiro Hara No130) and AY583325 (isolate ATCC200796) correspond to *M. corallinus* (Roberge) Diederich and D. Hawksw.

Key to the known species of *Marchandiomyces*

1. Colonies made of minute pinkish to red bulbils, basidiomata absent……………‥……………………………………2
1. Basidiomata corticioid, bulbils unknown……………….…3
2. On lichen thalli……….…‥…………………***M. corallinus***
2. On decorticated wood…………………………***M. lignicola***
3. Basidiomata visible as tiny patches around lenticels on *Quercus* branches………. ……‥………***M. aurantioroseus***
3. Basidiomata inside wood, detectable only as faint pink area on decorticated wood…………. ………. …………………4
4. Basidiospores allantoid, on conifers (*Juniperus, Pinus*), in France………………. ………***M. allantosporus*** sp. nov.
4. Basidiospores widely ellipsoid, on *Quercus brantii*, in Iran…………………………………***Marchandiomyces*** sp.

### Lichenicolous Taxa in Corticiaceae

Several species of Corticiaceae are known to grow on lichens. Some of them are very common and kill entire corticolous lichen populations. Thus, they play an important role in the dynamics of epiphytic lichen communities. Until recently, these fungi were poorly known and misunderstood.

The best known and most widespread species is *Marchandiomyces corallinus*. This fungus was initially described as *Illosporium corallinum* Roberge in a genus typified on *Illosporium carneum* Fr., the sporodochial state of *Pronectria* species (Hypocreales, Ascomycota). In his revision of lichenicolous hyphomycetes, Hawksworth (1979) included the species in *Illosporium*, but noted that the morphology and anatomy strongly differ from that of *I. carneum*. He further explained that the pinkish and the orange populations might represent distinct species. Diederich (1990) described the new genus *Marchandiomyces* to accommodate *M. corallinus*, and later Etayo and Diederich (1996) distinguished the orange-colored populations as *M. aurantiacus* (Lasch) Diederich and Etayo.

Sikaroodi et al. (2001) demonstrated that both *Marchandiomyces* species belong to the Basidiomycota, whereas *Illosporium carneum* is related to hypocrealean fungi. DePriest et al. (2005) described a third species of *Marchandiomyces*, the non-lichenicolous *M. lignicola*, and suggested that the genus belongs to a sister clade of Corticiaceae. Lawrey et al. (2007) included these species in Corticiales, while Lawrey et al. (2008) were the first to include them in Corticiaceae. Meanwhile, several lichenicolous, lignicolous, or foliicolous *Marchandiomyces* species were described, of which some were eventually transferred to *Laetisaria* by Diederich et al. (2018), whereas *M. aurantiacus* was transferred by Hawksworth and Henrici (2015) to *Erythricium* as *E. aurantiacum*.

Currently, five lichenicolous species of Corticiaceae are known. (1) *Erythricium aurantiacum* is extremely abundant in corticolous Xanthorion lichen communities. It seasonally invades entire corticolous *Physcia* populations, kills the host thalli, leaving behind thin, whitish cortical remnants. It is not strictly host specific and occasionally grows on hosts belonging to other taxonomic groups. *Erythricium aurantiacum* is extremely rare in saxicolous lichen communities. (2) *Laetisaria lichenicola* is another virulent parasite of *Physcia adscendens* and *P. tenella* lichen populations, sometimes spreading over adjacent thalli of *Xanthoria parietina*, widespread in Europe, but much rarer. *Laetisaria lichenicola* kills the host thallus in a similar manner, reducing it to thin, shiny, varnish-like thalli; remnants of host thalli often fuse together, sometimes resulting in mixed *Physcia*/*Xanthoria* thalli. (3) *Marchandiomyces corallinus* is widespread and frequently collected, but by far less abundant than *E. aurantiacum*. It is known from over 40 host genera, including saxicolous and terricolous lichens. It nevertheless has a strong preference for some specific hosts, especially *Parmelia saxatilis* and *P. sulcata*. It usually kills individual host thalli, but does not affect entire lichen populations. (4) *Laetisaria buckii* resembles a small *M. corallinus*, is rarely collected in the USA, and is not host specific (Diederich and Lawrey, 2007). (5) The recently discovered and described *Corticium silviae* seems to be a specific parasite of *Thamnolia vermicularis* in the Alps (Diederich et al., 2018).

Some of these lichenicolous fungi, especially *M. corallinus* and *L. buckii*, only occur in the asexual state, producing minuscule pink or orange bulbils 70–250 μm diam (Figure 1). In *E. aurantiacum*, in addition to the common bulbil state, a rare sexual state has also been reported (Diederich et al., 2003). *Laetisaria lichenicola* and *C. silviae*, on the contrary, are always sexual, without known bulbil state. Basidiomata of *Erythricium aurantiacum* (orange) and of *Laetisaria lichenicola* (pink to coral) are small, floccose, rarely reaching 1 cm in diam., with an indeterminate margin. In both species, c. 0.5 μm large dolipores are easily visible by light microscopy. Basidiomata of *Corticium silviae* are pale pink, effused, thin, almost concolorous to the host thallus.

Interestingly, in addition to these lichenicolous species, the genus *Lawreymyces* Lücking and Moncada was described for seven endolichenic species, i.e., fungi growing asymptomatically inside lichen thalli (Lücking and Moncada, 2017). However, since these species are known only from DNA sequences obtained from lichen thalli—no cells of them have ever been observed and no descriptions or illustrations of morphological features were provided—the genus and the species names are unfortunately invalid, following ICN 38.1 (a), 40 (Ex. 6) (Barrie et al., 2012).

### Non-lichenicolous Taxa Known Only From the Asexual State

The three monotypic genera *Basidiodesertica, Giulia*, and *Disporotrichum*, as well as the species *Tretopileus sphaerophorus* (Berk. and M. A. Curtis) S. Hughes and Deighton are the non-lichenicolous taxa in Corticiaceae known only from their asexual state, and all are saprotrophic. Among these, *Giulia* is coelomycetous, producing pycnidia, while the rest of the taxa are hyphomycetous, with synnemata (*Basidiodesertica* and *T. sphaerophorus*) or without synnemata (*Disporotrichum*). In the absence of molecular data, it would have been impossible to find a link between these asexual forms and the basidiomycete family Corticiaceae.

*Basidiodesertica*, typified with *B. hydei* Maharachch., Wanas. and Al-Sadi, was recently described by Maharachchikumbura et al. (2021) from Oman deserts, inhabiting dead plant leaves. It is characterized by a pink stroma at the substrate surface, from which cylindrical to subulate synnemata arise, and by producing brown multicellular conidia. In the Corticiaceae phylogeny (Figure 2), *Basidiodesertica* together with *T. sphaerophorus* and *Giulia* form a well-supported clade sister to the genus *Corticium*.

*Tretopileus* contains three species, of which *T. sphaerophorus* is the only species for which sequence data is available (Jayawardena et al., 2019). The placement of *T. sphaerophorus* in the family Corticiaceae was shown by Ghobad-Nejhad et al. (2010). Morphologically, it produces synnemata with a capitate bulbil-like head proliferating about seven times to yield new bulbils. The species seems to be widely distributed (Okada et al., 1998).

The genus *Giulia* with the single species *G. tenuis* (Sacc.) Tassi ex Sacc. and D. Sacc. is known from Thailand and Italy, and produces black pycnidia and hyaline, appendaged conidia (Li et al., 2020). It was shown to belong to Corticiales by Rungjindamai et al. (2008).

*Disporotrichum* typified with *D. dimorphosporum* (Arx) Stalpers is characterized by dimorphic conidia and hyphae bearing verticillate clamps. Its phylogenetic position within Corticiales was recently shown by Gruhn and Ghobad-Nejhad (2021). Its nutritional mode is not yet known, as the species has been isolated from potato meal, air contaminant, plant medicine tablet, and horse skin (Stalpers, 1984). As shown in the Corticiaceae phylogram in Figure 2, *Disporotrichum* takes a position distant from *Basidiodesertica, Giulia*, and *Tretopileus*.

## Conclusions

In the current study, Corticiaceae is shown to be a relatively small family currently with ten monophyletic genera. Despite its size, it encompasses fungi amazingly different in their morphology and ecology, from saprotrophic corticioid species, to lichenicolous, bulbil-forming, and also hyphomycetous or coelomycetous taxa. An interesting feature of the family is a high tendency to form asexual structures, either alone or together with basidiomata. From a nomenclatural point of view, several species have dual names, for sexual and asexual forms, which were eventually united upon the recent one-fungus one-name paradigm. Our results highlight the need for more studies on functional attributes and the roles that members of the Corticiaceae play in ecosystems. It is possible that the evolutionary flexibility of ecological features such as substrate variability, reproduction forms, and nutritional modes diversity may have an impact on species diversification in the Corticiaceae. Consequently, upon time, we expect the family to expand in its size with the inclusion of some additional members, perhaps with further novel morphological or ecological features. In this sense, Corticiaceae might be a unique fungal family whose heterogeneous nature necessitates multidisciplinary collaboration across and beyond mycology.

## Data Availability Statement

The datasets presented in this study can be found in online repositories. The names of the repository/repositories and accession number(s) can be found in the article/Supplementary Material.

## Author Contributions

MG-N developed the original idea. MG-N and EL performed the experiments. MG-N and RN analyzed the molecular data. MG-N, KN, PD, RN, and MR drafted the manuscript. MG-N, KN, and PD performed morphological examinations. MG-N and JG performed field work and provided specimens. All authors have read the final manuscript and approved it.

## Conflict of Interest

The authors declare that the research was conducted in the absence of any commercial or financial relationships that could be construed as a potential conflict of interest. The handling editor RJ declared a past co-authorship with the authors MG-N, PD, and RN.

## Publisher's Note

All claims expressed in this article are solely those of the authors and do not necessarily represent those of their affiliated organizations, or those of the publisher, the editors and the reviewers. Any product that may be evaluated in this article, or claim that may be made by its manufacturer, is not guaranteed or endorsed by the publisher.

## References

[B1] Anderson J. B. Stasovski E. (1992). Molecular phylogeny of Northern hemisphere species of *Armillaria*. Mycologia 84, 505–516. 10.1080/00275514.1992.12026170

[B2] Ariyawansa H. A. Hyde K. D. Jayasiri S. C. Buyck B. Chethana K. W. T. Cui Y. Y.. (2015). Fungal diversity notes 111–246 - Taxonomic and phylogenetic contributions to fungal taxa. Fungal Diver. 75, 27–274. 10.1007/s13225-015-0346-5

[B3] Barrie F. R. Buck W. R. Demoulin V. Greuter W. Hawksworth D. L. Herendeen P. S.. (2012). International Code of Nomenclature for algae, fungi and plants (Melbourne Code), in ed McNeill J. (Königstein: Koeltz Scientific Books).

[B4] Binder M. Hibbett D. S. Larsson K-H. Larsson E. Langer E. Langer G. (2005). The phylogenetic distribution of resupinate forms across the major clades of mushroom-forming fungi (homobasidiomycetes). System. Biodivers. 3, 113–157. 10.1017/S1477200005001623

[B5] Binder M. Larsson K. H. Matheny B. P. Hibbett D. S. (2010). Amylocorticiales ord. nov. and Jaapiales ord. nov.: early diverging clades of Agaricomycetidae dominated by corticioid forms. Mycologia 102, 865–880. 10.3852/09-288 20648753

[B6] Boidin J. Gilles G. (1998). Contribution à l'étude des genres *Dendrocorticium, Dendrodontia* et *Dentocorticium* (*Basidiomycotina*). Cryptogam. Mycol. 19, 181–202.

[B7] Boidin J. Lanquetin P. (1984). Répertoire des données utiles pour effectuer les tests d'intercompatibilité chez les basidiomycètes. III Aphyllophorales non porées. Cryptogam. Mycol. 5, 193–245.

[B8] Boidin J. Lanquetin P. (1995). Sur quelques Corticiés (Basidiomycotina) de l'Éthiopie. Cryptogam. Mycol. 16, 85–99.

[B9] Boidin J. Lanquetin P. (1997). Répertoire des données utiles pour effectuer les tests d'intercompatibilité chez les basidiomycètes VII. Aphyllophorales non porées (deuxième supplément). Cryptogam. Mycol. 18, 9–18.

[B10] Boidin J. Terra P. Lanquetin P. (1968). Contribution à la connaissance des caractères mycéliens et sexuels des genres *Aleurodiscus, Dendrothele, Laeticorticium* et *Vuilleminia*. Bull. Soc. Mycol. France 84, 53–84.

[B11] Burdsall H. H. Gilbertson R. L. (1982). New species of Corticiaceae (Basidiomycotina, Aphyllophorales) from Arizona. Mycotaxon 15, 333–340.

[B12] Burt E. A. (1926). The Thelephoraceae of North America I–XV. Annals of the Missouri Botanical Garden. New York, NY: Hafner Publishing Company. 921 p.

[B13] Castresana J. (2000). Selection of conserved blocks from multiple alignments for their use in phylogenetic analysis. Molec. Biol. Evol. 17, 540–552. 10.1093/oxfordjournals.molbev.a026334 10742046

[B14] Chevallier F. F. (1826). Flore Générale des Environs de Paris 1, i–xxiv. Paris: France: Ferra Jeune. 674 p.

[B15] Coetzee M. Wingfield B. D. Wingfield M. J. (2018). *Armillaria* root-rot pathogens: species boundaries and global distribution. Pathogens 7:83. 10.3390/pathogens7040083 30356027 PMC6313743

[B16] Cunningham G. H. (1963). The Thelephoraceae of Australia and New Zealand. Bull. New Zealand Dept. Sci. Indust. Res. 145, 1–359.

[B17] DePriest P. T. Sikaroodi M. Lawrey J. D. Diederich P. (2005). *Marchandiomyces lignicola* sp. nov. shows recent and repeated transition between a lignicolous and a lichenicolous habit. Mycol. Res. 109, 57–70. 10.1017/S0953756204001601 15736863

[B18] Diederich P. (1990). New or interesting lichenicolous fungi. 1. Species from Luxembourg. Mycotaxon 37, 297–330.

[B19] Diederich P. Lawrey J. (2007). New lichenicolous, muscicolous, corticolous and lignicolous taxa of *Burgoa* s.l. and *Marchandiomyces* s.l. (anamorphic Basidiomycota), a new genus for *Omphalina foliacea*, and a catalogue and a key to the non-lichenized, bulbilliferous basidiomycetes. Mycol. Prog. 6, 61–80. 10.1007/s11557-007-0523-3

[B20] Diederich P. Schultheis B. Blackwell M. (2003). *Marchandiobasidium aurantiacum* gen. et sp. nov., the teleomorph of *Marchandiomyces aurantiacus* (Basidiomycota, Ceratobasidiales). Mycol. Res. 107, 523–527. 10.1017/S0953756203007639 12884948

[B21] Diederich P. Zimmermann E Sikaroodi M. Ghobad-Nejhad M. Lawrey J. D. (2018). A first lichenicolous *Corticium* species (Corticiaceae, Corticiales), described from *Thamnolia* in Switzerland. Bull. Soc. Natural. Luxemb. 120, 49–56.

[B22] Donk M. A. (1963). The generic names proposed for Hymenomycetes. XIII. Additions and corrections to parts I–IX, XII (Conclusion). Taxon 12, 153–168. 10.2307/1216184

[B23] Donk M. A. (1964). A conspectus of the families of Aphyllophorales. Persoonia 3, 199–324.

[B24] Duhem B. (2011). Notule 2. Des *Corticium* minimalistes. Rhizomorphes 5, 3–5.

[B25] Duhem B. Michel H. (2006). Une nouvelle espèce de *Corticium* de la région méditerranéenne. Clé du genre *Corticium* sensu stricto. Bull. Soc. Mycol. France 122, 145–160.

[B26] Duhem B. Michel H. (2008). Une nouvelle espèce de *Corticium* s.s. à hyménium inclus dans le bois. Cryptogam. Mycol. 29, 113–119.

[B27] Duhem B. Michel H. (2009). Une espèce nouvelle de Corticium s.st. Etudes dans les genres *Dendrocorticium et Dentocorticium* (Basidiomycotina). Cryptogam. Mycol. 30, 161–179.

[B28] Eriksson J. (1958). Studies in the Heterobasidiomycetes and Homobasidiomycetes Aphyllophorales from Muddus National Park in North Sweden. Symb. Bot. Upsalienses 16, 1–172.

[B29] Eriksson J. Hjortstam K. (1970). *Erythricium*, a new genus of Corticiaceae (basidiomycetes). Sven. Bot. Tidskr. 64, 165–169.

[B30] Eriksson J. Hjortstam K. Ryvarden L. (1978). *Mycoaciella-Phanerochaete*, in The Corticiaceae of North Europe, Vol. 5, 889–1047.

[B31] Eriksson J. Ryvarden L. (1976). The Corticiaceae of North Europe. Vol. 4. Hyphodermella – Mycoacia. Oslo, Norway: Fungiflora.

[B32] Etayo J. Diederich P. (1996). Lichenicolous fungi from the western Pyrenees, France and Spain. II. More Deuteromycetes. Mycotaxon 60, 415–428.

[B33] Gardes M. Bruns T. D. (1993). ITS primers with enhanced specificity for basidiomycetes: application to the identification of mycorrhizae and rusts. Molec. Ecol. 2, 113–118. 10.1111/j.1365-294X.1993.tb00005.x 8180733

[B34] Ghobad-Nejhad M. Hallenberg N. (2011). *Erythricium atropatanum* sp. nov. (Corticiales) from Iran, based on morphological and molecular data. Mycol. Prog. 10, 61–66. 10.1007/s11557-010-0674-5

[B35] Ghobad-Nejhad M. Nilsson R. H. Hallenberg N. (2010). Phylogeny and taxonomy of the genus *Vuilleminia* (Basidiomycota) based on molecular and morphological evidence, with new insights into Corticiales. Taxon 59, 1519–1534. 10.1002/tax.595016

[B36] Gilbertson R. L. (1973). Notes on some corticioid lignicolous fungi associated with snowbanks in southern Arizona. Persoonia 7, 171–182.

[B37] Ginns J. Lefebvre M. N. L. (1993). Lignicolous corticioid fungi (Basidiomycota) of North America. Mycol. Mem. 19, 1–247.

[B38] Giraldo A. Crous P. W. Schumacher R. K. Cheewangkoon R. Ghobad-Nejhad M. Langer E. (2017). The Genera of Fungi—G3: *Aleurocystis, Blastacervulus, Clypeophysalospora, Licrostroma, Neohendersonia*, and *Spumatoria*. Mycol. Prog. 16, 325–348. 10.1007/s11557-017-1270-8

[B39] Greslebin A. G. Rajchenberg M. (2003). Diversity of Corticiaceae sens. lat. in Patagonia, southern Argentina. New Zealand J. Bot. 41, 437–446. 10.1080/0028825X.2003.9512861

[B40] Gruhn G. Ghobad-Nejhad M. (2021). A new species and a new combination in *Waitea* (Corticiales, Basidiomycota) and the phylogenetic affinity of *Disporotrichum*. Mycol. Iran. 7, 171–179. 10.22043/mi.2021.124280

[B41] Hallenberg N. (1986). Culture studies in Corticiaceae (Basidiomycetes). Windhalia 15, 9–18.

[B42] Hawksworth D. L. (1979). The lichenicolous hyphomycetes. Bull. Br. Museum Nat. Hist. Bot. Series 6, 183–300.

[B43] Hawksworth D. L. Henrici A. (2015). New resting places for *Laeticorticium quercinum* and *Marchandiobasidium aurantiacum*. Field Mycol. 16, 16–17. 10.1016/j.fldmyc.2015.01.007

[B44] He M. Q. Zhao R. L. Hyde K. D. Begerow D. Kemler M. Yurkov A.. (2019). Notes, outline and divergence times of Basidiomycota. Fungal Divers. 99, 105–367. 10.1007/s13225-019-00435-4

[B45] Herter W. G. F. (1910). Autobasidiomycetes. Kryptogamen-Flora der Mark Brandenburg 6, 1–192.

[B46] Hodkinson B. P. Moncada B. Lücking R. (2014). Lepidostromatales, a new order of lichenized fungi (Basidiomycota, Agaricomycetes), with two new genera, *Ertzia* and S*ulzbacheromyces*, and one new species, *Lepidostroma winklerianum*. Fungal Divers. 64, 165–179. 10.1007/s13225-013-0267-0

[B47] Hopple J. S. Jr. Vilgalys R. (1999). Phylogenetic relationships in the mushroom genus *Coprinus* and dark-spored allies based on sequence data from the nuclear gene coding for the large ribosomal subunit RNA: divergent domains, outgroups, and monophyly. Molec. Phylogen. Evol. 13, 1–19. 10.1006/mpev.1999.0634 10508535

[B48] Izumitsu K. Hatoh K. Sumita T. Kitade Y. Morita A. Tanaka C.. (2011). Rapid and simple preparation of mushroom DNA directly from colonies and fruiting bodies for PCR. Mycoscience 53, 396–401. 10.1007/S10267-012-0182-3

[B49] Jackson H. S. (1950). Studies of Canadian Thelephoraceae V. Two new species of *Aleurodiscus* on conifers. Can. J. Res. C 28, 63–77. 10.1139/cjr50c-003

[B50] Jayawardena R. S. Hyde K. D. Jeewon R. Ghobad-Nejhad M. Wanasinghe D. N. Liu N. G.. (2019). One stop shop II: taxonomic update with molecular phylogeny for important phytopathogenic genera: 26–50 (2019). Fungal Divers. 94, 41–129. 10.1007/s13225-019-00418-5

[B51] Larsen M. J. Gilbertson R. L. (1974). New taxa of *Laeticorticium* (Aphyllophorales, Corticiaceae). Can. J. Bot. 52, 687–690. 10.1139/b74-087

[B52] Larsen M. J. Gilbertson R. L. (1978). *Laeticorticium lombardiae* (Aphyllophorales, Corticiaceae): a newly recognized segregate from the *L. roseum* complex. Mycologia 70, 206–208. 10.1080/00275514.1978.12020221

[B53] Larsen M. J. Nakasone K. K. (1984). Additional new taxa of *Laeticorticium* (Aphyllophorales, Corticiaceae). Mycologia 76, 528–532. 10.1080/00275514.1984.12023874

[B54] Larsson K-H. (2007). Re-thinking the classification of corticioid fungi. Mycol. Res. 111, 1040–1063. 10.1016/j.mycres.2007.08.001 17981020

[B55] Larsson K-H. Larsson E. Kõljalg U. (2004). High phylogenetic diversity among corticioid homobasidiomycetes. Mycol. Res. 108, 983–1002. 10.1017/S0953756204000851 15506012

[B56] Lawrey J. D. Binder M. Diederich P. Molina M. C. Sikaroodi M. Ertz D. (2007). Phylogenetic diversity of lichen-associated homobasidiomycetes. Molec. Phylogen. Evol. 44, 778–789. 10.1016/j.ympev.2006.12.023 17291787

[B57] Lawrey J. D. Diederich P. Sikaroodi M. Gillevet P. (2008). Remarkable nutritional diversity in the *Corticiales*, including a new foliicolous species of *Marchandiomyces* (anamorphic *Basidiomycota, Corticiaceae*) from Australia. Am. J. Bot. 95, 816–823. 10.3732/ajb.0800078 21632407

[B58] Li G. J. Hyde K. D. Zhao R. L. Hongsanan S. Abdel-Aziz F. A. Abdel-Wahab M. A.. (2016). Fungal diversity notes 253–366 - Taxonomic and phylogenetic contributions to fungal taxa. Fungal Divers. 78, 1–237. 10.1007/s13225-016-0366-9

[B59] Li W-J. McKenzie E. H. C. Liu J-KJ. Bhat D. J. Dai D. Q. Camporesi E.. (2020). Taxonomy and phylogeny of hyaline-spored coelomycetes. Fungal Divers. 100, 279–801. 10.1007/s13225-020-00440-y

[B60] Lücking R. Moncada B. (2017). Dismantling *Marchandiomphalina* into *Agonimia* (Verrucariaceae) and *Lawreymyces* gen. *nov*. (Corticiaceae): setting a precedent to the formal recognition of thousands of voucherless fungi based on type sequences. Fungal Divers. 84, 119–138. 10.1007/s13225-017-0382-4

[B61] Madeira F. Park Y. M. Lee J. Buso N. Gur T. Madhusoodanan N.. (2019). The EMBL-EBI search and sequence analysis tools APIs in 2019. Nucleic Acids Res. 47, W636–W641. 10.1093/nar/gkz268 30976793 PMC6602479

[B62] Maharachchikumbura S. S. Wanasinghe D. N. Cheewangkoon R. Al-Sadi A. M. (2021). Uncovering the hidden taxonomic diversity of fungi in Oman. Fungal Divers. 106, 229–268. 10.1007/s13225-020-00467-1

[B63] Martín M. P. Högberg N. Llistosella J. (1999). *Macowanites messapicoides*, a hypogeous relative of *Russula messapica*. Mycol. Res. 103, 203–208. 10.1017/S0953756298007035

[B64] Martín M. P. Zhang L-F. Fernández-López J. Dueñas M. Rodríguez-Armas J. L. Beltrán-Tejera E.. (2018). *Hyphoderma paramacaronesicum* sp. *Nov. (*Meruliaceae, Polyporales, Basidiomycota), a cryptic lineage to *H. macaronesicum*. Fungal System. Evol. 2, 57–68. 10.3114/fuse.2018.02.05 32467888 PMC7225581

[B65] Matheny P. B. Wang Z. Binder M. Curtis J. M. Lim Y. W. Nilsson R. H.. (2007). Contributions of *rpb2* and *tef1* to the phylogeny of mushrooms and allies (Basidiomycota, Fungi). Mol. Phylogenet. Evol. 43, 430–451. 10.1016/j.ympev.2006.08.024 17081773

[B66] Miller M. A. Pfeiffer W. Schwartz T. (2010). Creating the CIPRES Science Gateway for inference of large phylogenetic trees, in Proceedings of the Gateway Computing Environments Workshop (GCE), 14 Nov. 2010, New Orleans, LA, 1–8.

[B67] Nobles M. K. (1937). Production of conidia in *Corticium incrustans*. Mycologia 29, 557–566. 10.1080/00275514.1937.12017224

[B68] Nylander J. A. A. (2004). MrModeltest v2. Program distributed by the author. Evolutionary Biology Centre, Uppsala University, Uppsala.

[B69] O'Donnell K. (1993). *Fusarium* and its near relatives, in The fungal holomorph: mitotic, meiotic, and pleomorphic speciation in fungal systematics, eds Reynolds D. R. Taylor J. W. (Wallingford: CAB International), 225–233.

[B70] O'Donnell K. Gueidan C. Sink S. Johnston P. R. Crous P. W. Glenn A.. (2009). A two locus DNA sequence database for typing plant and human pathogens within the *Fusarium oxysporum* species complex. Fungal Gen. Biol. 46, 936–948. 10.1016/j.fgb.2009.08.006 19715767

[B71] Okada G. Takematsu A. Gandjar I. Nakase T. (1998). Morphology and molecular phylogeny of *Tretopileus sphaerophorus*, a synnematous hyphomycete with basidiomycetous affinities. Mycoscience 39, 21–30. 10.1007/BF02461574

[B72] Pagel M. Meade A. (2017). BayesTraits v.3.0.1. Available online at: http://www.evolution.rdg.ac.uk (accessed March 1, 2021).

[B73] Patouillard N. T. (1900). Essai Taxonomique sur les Familles et les Genres des Hyménomycètes. Lons-le-Saunier, Duclume, Paris. 184 p.

[B74] Pattengale N. D. Alipour M. Bininda-Emonds O. R. P. Moret B. M. E. Stamatakis A. (2009). How many bootstrap replicates are necessary? Lect. Notes. Comput. Sci. 5541, 184–200 10.1007/978-3-642-02008-7_13 20377449

[B75] Roberts P. (2003). New British records. 234. *Waitea circinata* Warcup & P. H. B. Talbot. Mycologist 17:63. 10.1016/S0269-915X(07)60045-7

[B76] Ronquist F. Teslenko M. Van Der Mark P. Ayres D. L. Darling A. Höhna S.. (2012). MrBayes 3.2: efficient Bayesian phylogenetic inference and model choice across a large model space. System. Biol. 61, 539–542. 10.1093/sysbio/sys029 22357727 PMC3329765

[B77] Rungjindamai N. Sakayaroj J. Plaingam N. Somrithipol S. Jones E. G. (2008). Putative basidiomycete teleomorphs and phylogenetic placement of the coelomycete genera: *Chaetospermum, Giulia* and *Mycotribulus* based on nu-rDNA sequences. Mycol. Res. 112, 802–810. 10.1016/j.mycres.2008.01.002 18501576

[B78] Sikaroodi M. Lawrey J. D. Hawksworth D. L. DePriest P. T. (2001). The phylogenetic position of selected lichenicolous fungi: *Hobsonia, Illosporium*, and *Marchandiomyces*. Mycol. Res. 105, 453–460. 10.1017/S0953756201003768

[B79] Silvestro D. Michalak I. (2010). raxmlGUI: a graphical front-end for RAxML. Available online at: http://sourceforge.net/projects/raxmlgui/ (accessed August 5, 2021).

[B80] Stalpers J. A. (1978). Identification of wood-inhabiting Aphyllophorales in pure culture. Stud. Mycol. 16, 1–248.

[B81] Stalpers J. A. (1984). A revision of the genus *Sporotrichum*. Stud. Mycol. 24, 1–105.

[B82] Swofford D. L. (2021). PAUP v.4.a168. Available online at: http://paup.phylosolutions.com/ (accessed March 1, 2021).

[B83] Tamura K. Stecher G. Peterson D. Filipski A. Kumar S. (2013). MEGA6: molecular evolutionary genetics analysis version 6.0. Molec. Biol. Evol. 30, 2725–2729. 10.1093/molbev/mst197 24132122 PMC3840312

[B84] Thiers B. (2021). Index Herbariorum: A Global Directory of Public Herbaria and Associated Staff . New York Botanical Garden's Virtual Herbarium. Available online at: http://sweetgum.nybg.org/ih (accessed March 1, 2021).

[B85] White T. J. Bruns T. Lee S. Taylor J. W. (1990). Amplification and direct sequencing of fungal ribosomal RNA genes for phylogenetics, in PCR Protocols: A Guide to Methods and Applications, eds Innis M. A. Gelfand D. H. Sninsky J. J. White T. J. (New York, NY: Academic Press),315–322.

[B86] Wurzbacher C. Larsson E. Bengtsson-Palme J. Van den Wyngaert S. Svantesson S. Kristiansson E.. (2019). Introducing ribosomal tandem repeat barcoding for fungi. Molec. Ecol. Resource. 19, 118–127. 10.1111/1755-0998.12944 30240145

[B87] Zoller S. Scheidegger C. Sperisen C. (1999). PCR primers for the amplification of mitochondrial small subunit ribosomal DNA of lichen-forming ascomycetes. Lichenologist 31, 511–516. 10.1006/lich.1999.0220

