# Supplementary material for: Digging Up the Roots: Taxonomic and Phylogenetic Disentanglements in Corticiaceae s.s. (Corticiales, Basidiomycota) and Evolution of Nutritional Modes
Source: Front Microbiol. 2021 Aug 25;12:704802. doi: 10.3389/fmicb.2021.704802 (PMC8425454; doi:10.3389/fmicb.2021.704802)
Supplement: Supplementary file 1 [file Data_Sheet_1.docx]

**Supplementary Table 1.** List of species sampled in the phylogenetic analyses. Ex-type isolates/vouchers are marked with an asterisk (*). Newly generated sequences are given in bold.

| **Species** | **Isolate/voucher** | **ITS** | **nLSU** | **nSSU** | **mtSSU** | **IGS** |
| --- | --- | --- | --- | --- | --- | --- |
| *Australovuilleminia coccinea* | BCP5551 (PDD); MG74* | HM046875 | HM046930 | – | – | – |
| *Basidiodesertica hydei* | DST2020a_SQUCC15289* | MW077150 | MW077159 | MW077166 | – | – |
| *Bernardia incrustans* | HHB-12952-sp | – | AF518617 | AF518578 | AF518679 | – |
| *B. incrustans* | Duhem 3613 (PC); MG172 | **MW805860** | **MW805825** | – | – | – |
| *B. incrustans* | CBS17236 | MH855759 | MH867272 | – | – | – |
| *B. incrustans* | CBS17336 | MH855760 | MH867273 | – | – | – |
| *Corticium boreoroseum* | F36087 (S); MG42 | **MW805842** | **MW805816** | – | – | – |
| *C. boreoroseum* | F112635 (S); MG46 | **MW805845** | HM046919 | – | – | – |
| *C. boreoroseum* | F13990 (S); MG47 | **MW805846** | HM046920 | – | – | – |
| *C. canfieldii* | ERC-72-11 (CFMR); MG151* | **MW805850** | **MW805821** | – | – | **MW805804** |
| *C.* cf. *canfieldii* | CFMR DLL-2011026 | KJ140550 | – | – | – | – |
| *C. erikssonii* | F119127 (S); MG44 | **MW805843** | **MW805818** | – | – | – |
| *C. erikssonii* | Ghobad-Nejhad 3237; MG286 | KU213579 | – | – | – | – |
| *C. lombardiae* | HCFC 1568; MG146 | **MW805847** | **MW805820** | – | **MW805797** | **MW805801** |
| *C. lombardiae* | HCFC 1570; MG147 | **MW805848** | – | – | – | **MW805802** |
| *C. lombardiae* | HCFC 1569; MG148 | **MW805849** | – | – | – | **MW805803** |
| *C. lombardiae* | MA-Fungi 74936; MG189 | **MW805862** | **MW805826** | – | – | **MW805812** |
| *C. malagasoroseum* | PC0094401 (PC); MG165* | **MW805856** | **MW805822** |  | **MW805799** | **MW805807** |
| *C. meridioroseum* | Ghobad-Nejhad 4005; MG290 | KU213583 | – | – | – | – |
| *C. meridioroseum* | Ghobad-Nejhad 3207; MG289 | KU213582 | – | – | – | – |
| *C. meridioroseum* | Duhem 5286 (PC); MG156 | **MW805852** | – | – | – | **MW805805** |
| *C. meridioroseum* | 09.709 (PC); MG168 | **MW805857** | – | – | – | **MW805809** |
| *C. meridioroseum* | MA-Fungi 26179; MG191 | **MW805863** | – | – | – | **MW805813** |
| *C. meridioroseum* | MA-Fungi 26166; MG192 | **MW805864** | – | – | – | **MW805814** |
| *C. roseum* | Ghobad-Nejhad 2428; MG252 | **MW805872** | **MW805836** | – |  |  |
| *C. roseum* | Ghobad-Nejhad 2358; MG253 | **MW805873** | **MW805837** | – | – | – |
| *C. roseum* | Ghobad-Nejhad 2245; MG254 | **MW805874** | **MW805838** | – | – | – |
| *C. roseum* | Ghobad-Nejhad 2229; MG255 | **MW805875** | **MW805839** | – | – | – |
| *C. roseum* | Ghobad-Nejhad 2387(ICH); MG256 | **MW805876** | – | – | – | – |
| *C. roseum* | Niemelä 7759 (H); MG43 | GU590877 | **MW805817** | – | – | – |
| *C. roseum* | AFTOL1943 | – | EF537893 | – | – | – |
| *C. roseum* | CBS10452 | MH856944 | MH868469 | – | – | – |
| *C. roseum* | Kotiranta 22291 (H); MG41 | **MW805841** | **MW805815** | – | – | – |
| *C. roseum* | F119043 (S); MG45 | **MW805844** | **MW805819** | – | – | – |
| *C. roseum* | FCUG2558 | **MW805880** | – | – | – | – |
| *C. roseum* | FCUG2582 | **MW805881** | – | – | – | – |
| *C. silviae* | S. Feusi 05.06.2017* | MH520061 | MH520061 | – | – | MH520061 |
| *C. thailandicum* | Ghobad-Nejhad 3012 (ICH); MG242* | **MW805868** | **MW805831** | – | – | – |
| *Cytidia salicina* | Haikonen 24631 (H); MG49 | GU590881 | HM046921 | – | AF214458 (KCTC6997; CBS727.85) | – |
| *Dendrocorticium polygonioides* | CBS11056 | MH857528 | MH869064 | – | – | **MW805806** (MG158) |
| *D. roseocarneum* | CBS33066 | MH858810 | MH870449 | AF334910 (FPL1800) | AF334875 (FPL1800) | **MW805808** (MG166) |
| *Dendrominia dryina* | Duhem 5283 (PC); MG159 | JX892936 | JX892937 | – | – | – |
| *D. ericae* | Duhem 4840 (PC); MG162* | JX892938 | JX892939 | – | – | – |
| *D. maculata* | HHB10621 | – | AY586652 | – | – | – |
| *Disporotrichum dimorphosporum* | CBS 433.85 | MH861895 | MH873584 | – | – | – |
| *D. dimorphosporum* | CBS 610.71 | MH860288 | – | – | – | – |
| *D. dimorphosporum* | CBS 419.70* | MH859776 | MH871538 | – | – | – |
| *Erythricium atropatanum* | Ghobad-Nejhad 1238; MG58* | GU590876 | GU590880 | – | – | – |
| *E. aurantiacum* | CBS 128706 | MH864966 | MH876416 | – | – | – |
| *E. aurantiacum* | CBS 718.97 | AY583324 | AY583330 | DQ915460 (JL219-01; ATCC MYA 2502) | – | – |
| *E. aurantiacum* | JL391-10 | HQ168397 | HQ168397 | HQ168398 | HQ168388 | – |
| *E. hypnophilum* | M. Meyer (PC); MG169 | **MW805858** | **MW805823** | – | – | – |
| *E. hypnophilum* | M. Meyer -33 (PC); MG170 | **MW805859** | **MW805824** | – | – | – |
| *E. hypnophilum* | Haikonen 15143 (H); MG271 | **MW805878** | – | – | – | – |
| *E. laetum* | MG72 | GU590875 | GU590878 | – | – | – |
| *E. laetum* | MG73 | GU590874 | GU590879 | – | – | – |
| *E. laetum* | Saarenoksa 00795 (H); MG272 | – | **MW805840** | – | – | – |
| *E. salmonicolor* | BNR-KT-06 | EU435008 | AY672680 (CMW9580) | – | – | – |
| *E. salmonicolor* | Royal Delicious | KF029722 | KF029722 | – | – | – |
| *E. salmonicolor* | CBS16782 | – | AY672682 | – | – | – |
| *E. vernum* | RLG-7886 (CFMR); MG236 |  | **MW805827** | – | – | – |
| *E. vernum* | FP-133815 (CFMR); MG237* | **MW805865** | **MW805828** | – | – | – |
| *E. vernum* | FP-133814-Sp (CFMR); MG238 | **MW805866** | **MW805829** | – | – | – |
| *E. vernum* | FP-133817 (CFMR); MG239 | **MW805867** | **MW805830** | – | – | – |
| *E. vernum* | Ginns 10782; MG247 | **MW805869** | **MW805832** | – | – | – |
| *E. vernum* | Ginns 11684; MG248 |  | **MW805833** | – | – | – |
| *E. vernum* | FP-133816 (CFMR); MG240 | **MW805870** | **MW805834** | – | – | – |
| *E. vernum* | DAOM 172906; MG250 | **MW805871** | **MW805835** | – | – | – |
| *Giulia tenuis* | BCC13066 | – | EF589739 | EF589732 | – | – |
| *Gloeophyllum sepiarium* | CFMR Wilcox-3BB (reference material) | NR_119869 | NG_060630 | NG_064955 | U27041 | – |
| *Laetisaria agaves* | RLG-10805 (CFMR); MG152* | **MW805851** | – | – | – | – |
| *L. buckii* | ATCC MYA 2992; JL244-03* | – | DQ915472 | DQ915462 | HQ168392 | – |
| *L. culmigena* | ATCC 22523; clone 22523.12.20.07 | EU622849 | EU622848 | EU622847 | – | – |
| *L. culmigena* | CBS13669 | – | MH871008 | – | – | – |
| *L. endoxylon* | MA-Fungi 78580; MG186 | – | – | – | – | **MW805810** |
| *L. endoxylon* | MA-Fungi 81085; MG187 | **MW805861** | – | – | – | **MW805811** |
| *L. endoxylon* | Duhem 5279 (PC); MG157 | **MW805853** | – | – | – | – |
| *L. endoxylon* | Duhem 4765 (PC); MG160* | **MW805854** | – | – | **MW805798** | – |
| *L. endoxylon* | PC0095927; MG161 (paratype) | **MW805855** | – | – | – | – |
| *L. fuciformis* | NJ-2 Jackson | EU118639 (Hjm 18391) | AY293192 | AY293139 | AY293232 | – |
| *L. fuciformis* | CBS 18249 | MH856485 | MH868023 | – | – | – |
| *L. lichenicola* | CBS 128705; JL393-10* | NR_121484 | HQ168400 | HQ168399 | HQ168389 | – |
| *L. marsonii* | ATCC MYA 4210* | NR_164214 (EU622840) | EU622839 | EU622838 | HQ168395 | – |
| *L. nothofagicola* | JL-261-04* | DQ915474 | DQ915474 | DQ915466 | HQ168394 | – |
| *L. roseipellis* | CBS 299.82 | EU622846 | EU622844 | EU622845 | HQ168396 | – |
| *‘Lawreymyces palicei’* | Palice 4369* | AY542865 | AY542865 | – | – | – |
| *‘Lawreymyces palicei’* | Palice 2509 | AY542864 | AY542864 | – | – | – |
| *Leptocorticium tenellum* | MG143 | KU183719 | KU183720 | – | – | – |
| *Marchandiomyces allantosporus* | Duhem 5354 (PC); MG259* | **MW805877** | – | – | – | – |
| *M. aurantioroseus* | FCUG1151 | **MW805879** | – | – | – | – |
| *M. aurantioroseus* | FCUG1166 | KP864659 | HM046929 (FCUG 1171) | – | – | – |
| *M. corallinus* | ATCC MYA 3182 | AY583327 (JL12898) | AY583331 (JL12898) | DQ915464 | HQ168393 | – |
| *M. corallinus* | ATCCMYA1118 | AY583326 | – | – | – | – |
| *M. lignicola* | ATCC MYA 3674 | FJ172272 (ATCCMYA4208) | AY583332 (ATCCMYA299) | DQ915465 | HQ168391 | – |
| *M. lignicola* | ATCCMYA299* | AY583328 | – | – | – | – |
| *M. lignicola* | ATCCMYA835 | AY583329 | – | – | – | – |
| *Marchandiomyces* sp. | MG287 | KU213580 | – | – | – | – |
| *Punctularia strigosozonata* | CBS 345.34 | MH855559 | MH867064 | – | – | – |
| *P. strigosozonata* | AFTOL-ID 1248; HHB-11897-sp | DQ398958 | AF518642 | AF518586 | – | – |
| *Punctulariopsis cremeoalbida* | MG153; Burdsall 9616* | KR494275 | – | – | – | – |
| *P. efibulata* | MG150; Burdsall 8824* | KR494276 | KR494277 | – | – | – |
| *P. obducens* | Ryvarden 28131 (O); MG70* | HM046918 | HM046933 | – | – | – |
| *P. subglobispora* | FCUG2535* | HM046917 | HM046932 | – | – | – |
| *Tretopileus sphaerophorus* | JCM10092 | – | – | AB006005 | – | – |
| *Vuilleminia comedens* | T-583 | HM046880 (MG79) | AF518666 | AF518594 | AF518699 | – |
| *V. comedens* | MG86 | HM046882 (MG10) | HM046922 | – | – | **MW805800** (MG109) |
| *V. cystidiata* | FCUG 2596 | HM046909 | HM046923 (MG94) | – | – | – |
| *V. pseudocystidiata* | MG69* | HM046888 | HM046928 | – | – | – |
| *Waitea arvalis* | CBS 131.82* | EU622841; MH861489; NR_119689 | EU622842; MH873229 | EU622843 | HQ168390 | – |
| *W. circinata* | AFTOL-ID 1129 | DQ356414 (Rh 120232) | AY885164 | MT305028 (NSF46) | FJ440234 (YWK-178) | – |
| *W. circinata* | CBS47282 | MH861518 | MH873265 | MT305027 (NSF45) | FJ440232 (YWK-160) | – |
| *W. circinata* | CBS27338 | MH855962 | MH867457 | – | – | – |
| *W. circinata* | X-54 | KC176341 | KC176341 | MT305023 (NSF7) | FJ440221 (YWK-77) | – |
| *W. guianensis* | GGGUY13110 | MW449090 | MW449101 | MW449084 | – | – |

**Supplementary Table 2.** Mating types and nuclear behavior of mycelia in Corticiaceae.

| **Species** | **No. nuclei/spore** | **Mating type** | **Nuclear behavior** | **Spore print** | **Ref.** |
| --- | --- | --- | --- | --- | --- |
| *Bernardia incrustans* | ‒ | Heterothallic bipolar | Heterocytic | ‒ | Nobles (1937); Stalpers (1978) |
| *Corticium boreoroseum* | 2 | Heterothallic | Heterocytic | ‒ | Boidin and Lanquetin (1984) |
| *C. canfieldii* | ‒ | Heterothallic bipolar | ‒ | ‒ | Larsen and Gilbertson (1974) |
| *C. meridioroseum* | 2 | Heterothallic bipolar | Heterocytic | ‒ | Boidin et al. (1968); Boidin and Lanquetin (1984) |
| *C. minnsiae* | ‒ | Heterothallic bipolar | ‒ | pink | Jackson (1950) |
| *C. pini* | ‒ | Heterothallic bipolar | ‒ | pink | Jackson (1950); Boidin and Lanquetin (1984) |
| *C. roseum* | 2 | Homothallic/  Heterothallic bipolar | ‒/  Heterocytic | pink | Eriksson (1958); Jackson (1950); Boidin et al. (1968); Larsen and Gilbertson (1978); Boidin and Lanquetin (1984)* |
| *Erythricium laetum* | 1 | Supposedly homothallic | Holocoenocytic | ‒ | Boidin and Lanquetin (1997) |
| *E. laetum* | 2 | ‒ | ‒ | pink | Boidin and Lanquetin (1995) |
| *E. salmonicolor* | 2 | ‒ | ‒ | pink | Boidin and Lanquetin (1995) |
| *Marchandiomyces aurantioroseum* (= *M. quercinus*) | 2 | Supposedly homothallic | Holocoenocytic | ‒ | Hallenberg (1986) |

**Corticium lombardiae* was described as bipolar by Larsen and Gilbertson (1978). Boidin and Lanquetin (1984) stated it as heterocytic (as inedit data).
